# Cross-regional homeostatic and reactive glial signatures in multiple sclerosis

**DOI:** 10.1007/s00401-022-02497-2

**Published:** 2022-09-16

**Authors:** Tim Trobisch, Amel Zulji, Nikolas A. Stevens, Sophia Schwarz, Sven Wischnewski, Mikail Öztürk, Javier Perales-Patón, Maximilian Haeussler, Julio Saez-Rodriguez, Dmitry Velmeshev, Lucas Schirmer

**Affiliations:** 1 Division of Neuroimmunology, Department of Neurology, Medical Faculty Mannheim, Heidelberg University, Mannheim, Germany; 2 Institute for Physiology and Pathophysiology, Heidelberg University, Heidelberg, Germany; 3 Institute for Computational Biomedicine, Faculty of Medicine, Heidelberg University and Heidelberg University Hospital, BioQuant, Heidelberg, Germany; 4 Genomics Institute, University of California, Santa Cruz, Santa Cruz, CA USA; 5 Department of Neurology, University of California, San Francisco, San Francisco, CA USA; 6 Eli and Edythe Broad Center of Regeneration Medicine and Stem Cell Research, University of California, San Francisco, San Francisco, CA USA; 7 Department of Neurobiology, Duke University School of Medicine, Durham, NC USA; 8 Mannheim Center for Translational Neuroscience and Institute for Innate Immunoscience, Medical Faculty Mannheim, Heidelberg University, Mannheim, Germany; 9 Interdisciplinary Center for Neurosciences, Heidelberg University, Heidelberg, Germany

**Keywords:** Single-nucleus RNA-sequencing, Glial heterogeneity, Astrocytes, Oligodendrocytes, Lesion pathology

## Abstract

Multiple sclerosis (MS) is a multifocal and progressive inflammatory disease of the central nervous system (CNS). However, the compartmentalized pathology of the disease affecting various anatomical regions including gray and white matter and lack of appropriate disease models impede understanding of the disease. Utilizing single-nucleus RNA-sequencing and multiplex spatial RNA mapping, we generated an integrated transcriptomic map comprising leukocortical, cerebellar and spinal cord areas in normal and MS tissues that captures regional subtype diversity of various cell types with an emphasis on astrocytes and oligodendrocytes. While we found strong cross-regional diversity among glial subtypes in control tissue, regional signatures become more obscure in MS. This suggests that patterns of transcriptomic changes in MS are shared across regions and converge on specific pathways, especially those regulating cellular stress and immune activation. In addition, we found evidence that a subtype of white matter oligodendrocytes appearing across all three CNS regions adopt pro-remyelinating gene signatures in MS. In summary, our data suggest that cross-regional transcriptomic glial signatures overlap in MS, with different reactive glial cell types capable of either exacerbating or ameliorating pathology.

**Supplementary Information:**

The online version contains supplementary material available at 10.1007/s00401-022-02497-2.

## Introduction

Multiple sclerosis (MS) is a multifocal progressive inflammatory disease of the central nervous system (CNS) characterized by demyelination, astrogliosis and varying levels of cell type vulnerability and reactivity during lesion development [18, 43, 46]. Disease presentation strongly correlates with the spatial dissemination of lesions throughout CNS which includes spinal cord, cerebellum and cerebrum among other areas [43]. Hence, a comprehensive understanding of the disease demands not only to assess the roles of heterogeneous cell populations involved in disease pathology but also to understand the pathological mechanisms underlying lesion formation in different CNS compartments. It has been shown that neurons and subtypes of glial cells differ between cortical areas and show variable cell type-dependent levels of damage in MS [48, 52]. Moreover, extensive research has been conducted to investigate the roles of astrocytes [30], oligodendrocytes [22, 40] and neurons [48] in MS; however, these studies focused primarily on individual CNS regions such as the spinal cord or the cerebrum. Hence, it is unclear whether gene expression reflecting cell type damage and reactivity in MS differs between CNS regions and subregions, such as gray and white matter.

To assess cell type diversity and reactivity in MS across different CNS regions, we performed droplet-based single-nucleus RNA-sequencing (snRNA-seq) from well-characterized tissue samples comprising cerebellar, spinal cord and leukocortical samples [48] obtained from MS and matched control subjects. Following data integration, we investigated glial cell heterogeneity between CNS regions in an anterior–posterior fashion and observed regionally diverse patterns of control glial subtypes, including astrocytes and oligodendrocytes. Next, we aimed at identifying changes in MS lesion pathology on different hierarchical levels. First, we identified global MS gene expression changes without respect to different CNS regions among various neuroglial subtypes, including the 17q.21.31 locus, which has been linked to a genetic risk for neurological diseases. Next, we assessed distinct glial subtypes and identified upregulation of glial reactivity genes with an emphasis on astrocytes and oligodendrocytes shared between CNS regions in MS. In particular, we identified an endogenous mechanism associated with remyelination in a subtype of white matter oligodendrocytes, which becomes activated in white matter MS lesion tissue areas.

## Methods

### Origin of postmortem human tissue samples

Postmortem human tissue samples from a total of 41 donors (21 female and 20 male) were provided by the UK Multiple Sclerosis Tissue Bank at Imperial College, London via prospective donor scheme following ethical approval by the National Research Ethics Committee in the UK (08/MRE09/31). Samples were obtained from 21 donors without evidence for pathological abnormalities within the examined tissue areas and from 20 donors who were diagnosed with multiple sclerosis (MS). Additional clinical and pathological details about control and MS donors as well as quality control of the samples are provided (Suppl. Table 1, Online Resources).

### Sample selection

Integrity of mRNA was used as a sample selection criterion, and only samples with a high quality of tissue RNA were included. We prepared 100-µm thick sections on a CM3050S cryostat (Leica) to obtain 10 mg tissue to isolate RNA, which was done using TRIzol (Thermo Fisher) and Qiagen RNAeasy Kit following manufacturer’s recommendations. RNA integrity was measured on an Agilent 2100 Bioanalyzer using the RNA 6000 Pico Kit (Agilent) according to the manufacturer’s instructions. Samples with RNA integrity number (RIN) ≥ 6.5 were selected for further snRNA-seq analysis.

### Histopathological assessment

Histopathological assessment was carried out using immunohistochemistry (IHC) for CD45, IBA1, CD68, CD3 and MOG as described previously [48], as well as Luxol fast blue and hematoxylin/eosin staining. Following primary antibodies were used: mouse anti-MOG (clone 8-18C5, 1:1,000, Millipore Sigma, RRID AB_1587278), mouse anti-CD45 (clone H130, 1:200, Biolegend, RRID AB_314390), rabbit anti-IBA1 (polyclonal, 1:500, Wako, RRID AB_839504), mouse anti-CD68 (clone 514H12, 1:100, Bio-Rad, RRID AB_2074721) and rat anti-CD3 (clone CD3-12, 1:200, Biorad, RRID AB_321245). Primary antibodies were labeled using cross-adsorbed secondary goat IgG antibodies (H + L) against different species (mouse, rabbit and rat) tagged to various fluorophores (Alexa Fluor Plus 488, 555 and 647, Thermo Fisher Scientific). All included MS patient tissue samples harbored demyelinated lesions with adjacent lesion rim and normal-appearing gray and white matter areas of varying inflammatory activity. Lesion areas identified according to MOG IHC were then further classified in acute, chronic-active and chronic-inactive lesions. Acute lesions had an indistinct rim and showed active demyelination with presence of myeloid foam cells (MOG^+^/CD45^+^/CD68^high^) in lesion center areas (Suppl. Fig. 1a, Online Resources) and perivascular cuffing of T cells (CD45^+^/CD3^+^) and macrophages (CD45^+^/IBA1^low^/CD68^high^) (Suppl. Fig. 1d Online Resources). Chronic active lesions were classified by a demarcated demyelinated lesion center and a hypercellular lesion rim with increased presence of macrophages and activated microglia (CD45^+^/IBA1^high^/CD68^low^) (Suppl. Fig. 1a-d, Online Resources). Inactive lesions demonstrated fully demyelinated lesion centers with absence of macrophages; in normal-appearing and periplaque lesion areas we observed an abundance of ramified microglia (CD45^+^/IBA1^high^) (Suppl. Fig. 1d, Online Resources) [28, 47].

### Preparation of cDNA libraries, sequencing and expression matrix generation

Nuclei from the selected samples were isolated using sucrose-gradient ultracentrifugation according to previous protocols [48, 55]. Following isolation, nuclei were diluted to a final concentration of 2,000 nuclei per μl and loaded on the 10 × Chromium single-cell expression platform (v2 chemistry) for cDNA library preparation aiming at a recovery rate of 4,000 nuclei per sample. Prepared cDNA libraries were sequenced on an Illumina HiSeq 2500 sequencing system aiming for a sequencing depth of 60,000 reads per nucleus. Expression matrices for each sample were generated with Cell Ranger Count v3.0.2 by performing alignment of sequencing data against a custom-built GRCh38 pre-mRNA reference transcriptome. The custom-built reference transcriptome was modified to allow additional capture of intronic reads originating from pre-mRNA transcripts. The final expression matrix, comprised of all 20 samples, was generated by merging single expression matrices using Cell Ranger Aggregate v3.0.2. Merging was performed without sequencing-depth normalization.

### Quality control and doublet filtration

As a quality control criterion, in downstream analysis we included only nuclei containing at least 250 genes and 400 counts (Suppl. Fig. 2a, b, Online Resources). Nuclei were further filtered in a way that 99% of the nuclei included in downstream analysis had less than 5% of mitochondrial genes (Suppl. Fig. 2c, Online Resources). In addition, doublets were removed using scDblFinder v1.4.0 [16] with default parameters except the dbr = 0.05 which was adjusted to accommodate higher doublet rates for nuclei as compared to cells [55].

### Normalization, scaling and variable features selection

The filtered expression matrix was processed using Seurat (v3.2.3) SCTransformation [51], a regularized negative binomial regression-based model, specifically developed for unique molecular identifiers (UMI) based data and including normalization, scaling and variable feature selection. SCTransform() was first performed with default parameters for the analysis of each region separately. For normalization of the integrated data set, SCTransform() was performed with variable.features. n = 5000.

### Dimensionality reduction and selection of principal components

Principal component analysis (PCA) was done using Seurat [51] RunPCA() calculating the top 50 principal components (PCs). For the selection of significant PCs to include, we used unbiased criteria. First, we selected the PC cutoff so that the cumulative variance explained of all prior PCs is more than 90%. Then, we determined the second PC cutoff so that the variance explained of consecutive PCs is less than 10%. Finally, number of significant PCs was taken as a minimum of above-mentioned metrics. Selecting PCs in this way avoids user subjectivity and ensures consistent analysis.

### Data integration and batch removal

To ensure clustering based on cell types and mitigate clusters driven by single samples, data were integrated using Harmony v1.0.0 [27], where each sample was treated as a batch. Built-in function RunHarmony() was run on the SCTransform corrected assay, using default parameters except setting parameter max.iter.harmony = 30. For selecting significant harmony components (corrected PCs), the same metrics were used as for selecting significant PCs described above, except for the integrated data where 31 harmony components were chosen.

### Clustering and visualization

Visualization and clustering were done in Seurat [51] following official vignette by developers. In brief, we used built-in functions RunUMAP(), FindNeighbors() and FindClusters() on harmony [27] corrected PC embeddings using default parameters, except setting resolution to 2.5.

### Subclustering of cell types

For cell subtype identification, sub-clustering was performed. Briefly, the cluster of interest was subset and PCA was rerun on normalized data, followed by selection of significant PCs, data integration with Harmony [27], clustering and visualization as previously described.

### Cluster markers and cell type annotation

Cluster markers were identified using function findMarkers() from scran v1.18.5 package [34] with the default parameters except setting pval.type = "all" (Suppl. Table 2, Online Resources). Clusters were then annotated based on known marker genes, which showed significant expression levels in the corresponding cluster.

### Analysis of cell population subsets

For subclustering of distinct populations like astrocytes or oligodendrocytes, we used in each case a subset of nuclei of the integrated data set, which was annotated as the cell type of interest based on its gene expression. Then, we performed the same analysis workflow as described above only for the respective subtype. In general, the resolution parameter was here reduced as we analyzed only one cell type.

### Cell correlation heatmap

To calculate the correlation between glial cell type expression profiles, gene expression of glial cell types was averaged for each cell type using the SCTransform corrected expression data. Then, Pearson correlation analysis was performed on the averaged expression profiles.

### Differential gene expression analysis

For the differential gene expression (DGE) analysis, the DESeq2 [33] pseudobulk approach was used. Briefly, for each sample raw counts were summed up and used as input for DESeq2. As gene filtering criterion, we included only genes having at least 2 counts in a minimum of 30 cells. After summing up gene counts, we introduced another filtering step excluding genes with less than 10 counts in at least 2 samples. For further quality control, plotPCA() function was used verifying that the main source of variance is explained by condition. Based on the results, clear outlier samples were excluded, which was never more than one. Within DESeq2, a standardized workflow including Wald test was used while controlling for sex differences. To better handle log2 fold changes, we used the apeglm shrinkage estimator v1.12.0 [58]. In case of DGE analysis between CNS regions, pairwise comparisons were calculated between each region. Intersection of these pairwise comparisons were taken as region-specific DEGs.

### Functional enrichment and semantic similarity analysis

Functional enrichment analysis of differentially expressed gene sets was performed using ClusterProfiler v3.18.0 implementation of GO terms [57]. Mitochondrial encoded and nuclear encoded ribosomal transcripts were excluded prior to analysis. As a background list, all genes which are expressed in the investigated cell type were used. Using bitr() function, we transferred gene symbols to Entrez gene IDs. As a database, we used R package org.Hs.eg.db v3.12.0 [6]. Genes without corresponding Entrez ID were excluded. GO term enrichment was then calculated with enrichGO() function using the biological processes database, *p* and *q* value cutoff of 0.05 and Benjamini–Hochberg procedure for adjusted *p* values. For semantic similarity analysis of gene clusters, we used R package GOSemSim v2.16.1 [56]. Correlation was calculated between each gene cluster, whereas one cluster consists of genes which were identified as differentially expressed and contribute to one of the previous identified GO terms. mclusterSim() function was used with biological processes of org.Hs.eg.db as database, the Wang method for calculation and Best-Match Average strategy (BMA) as combination method. After calculation, correlation matrix was visualized using pheatmap (R package version 1.0.12., https://CRAN.R-project.org/package=pheatmap) and grouped into clusters by cutting the dendrogram at height 3 to retrieve biological meaningful clusters.

### Transcription factor activity

For calculating transcription factor (TF) activity, R package dorothea v1.2.0 [15] was used. As input, normalized data derived from SCTransform was used. In addition, for technical reasons due to high memory demands with large datasets, the dataset was split into 5 random parts with an even sampling of cells from each cluster, then TF activity was calculated on single cells for each part separately using regulons with confidence intervals A, B, C [20]. Finally, single-cell TF activities were merged after correcting technical batches introduced by splitting the dataset using ComBat() function of package sva v3.38.0 [29].

### Cell–cell communication analysis

For the cell–cell communication (CCC) analysis, R package LIANA v0.1.5 [13] was used. Ligand receptor pairs were inferred on the normalized expression profiles using consensus rank from 5 different methods (CellPhoneDBv2, NATMI, Connectome, SingleCellSingleR, iTALK) which were run against the Omnipath resource. The Consensus rank is generated across all methods using Robust Rank Aggregation [25].

### Trajectory inference and pseudotime DGE analysis

Pseudotime trajectory analysis was done using R package slingshot v1.8.0 [50]*.* The trajectory was calculated based on UMAP embeddings of the corresponding Seurat object in unsupervised manner setting neither start- nor endpoint. The Seurat objects for subsets of oligodendrocytes consisted of same cells which were used for DGE analysis and were processed as described above except skipping RunHarmony() function to avoid losing differences of control and MS nuclei in UMAP embeddings. Differential gene expression analysis was carried out with R package tradeSeq v1.4.0 [54]. Analysis was done following the official tutorial by the developers using the diffEndTest() function. Only genes with more than 1 count in at least 100 cells in the normalized and scaled assay were included. Based on evaluateK() we selected nknots = 7 for calculation. For heatmap visualization we used a subset of all significantly differentially expressed genes, selecting only genes with high expression level (waldStat > 10) which were included in the SCTransform corrected assay of the two datasets containing *SLC5A11* oligodendrocytes from cerebrum and spinal cord, respectively.

### Fluorescence multiplex in situ RNA hybridization

For single-molecule fluorescence in situ hybridization (smFISH) validation we used frozen human cryosections (for slide preparation see section *Histopathological assessment*). Staining was performed on a representative selection of samples using ACD RNAscope 2.5 HD Red and Multiplex Fluorescent V2 assays. Slides were directly transferred from − 80 °C into 4% PFA for fixation; first 15 min at 4 °C, then 2 h at room temperature. After fixation, slides were incubated with H_2_O_2_ for 10 min and then boiled in Target Retrieval solution (ACD) for 5 min. Following a washing step in dH_2_O slides were dehydrated in 100% EtOH before protease treatment (Protease IV, ACD, 30 min at room temperature). Target probes (C1–C3) were then mixed and incubated on slides for 2 h at 40 °C in a specific RNAscope hybridization oven (ACD). The following human RNAscope assay probes were used: *LINC00685* (C1), *ADGRV1* (C1), *CPAMD8* (C1), *GRIA1* (C1), *SLC5A11* (C1), *CHRM5* (C1), *AQP4* (C2), *SYT1* (C2), *LINC01608* (C2), *MAG* (C2), *PITPNC1* (C3), *PLP1* (C3) and *GNA14* (C3). Slides were then washed and kept in 5X SSC overnight. Next day, amplification and probe channel detection steps were performed following manufacturer’s recommendation. In case of the multiplex smFISH assay, TSA Plus Cyanine 3 (Cy3, excitation maximum 554, emission maximum 568), TSA Plus Cyanine 5 (Cy5, excitation maximum 649, emission maximum 666) and TSA Plus Fluorescein (excitation maximum 490, emission maximum 525) detection kits (Akoya Biosciences) were used as fluorophores for probe labeling. Slides were mounted with ProLong Gold antifade reagent (Thermo Fisher Scientific).

### Microscopy, image acquisition and statistical analysis

Brightfield images were taken using a Leica DMi 8 microscope with a Leica DFC7000 GT camera. Confocal images were taken using a Leica TCS SP8, a Nikon AX R microscope with a Nikon Plan Apo λ 40 × NA 0.95 objective and a Nikon A1 with a Nikon Plan Fluor 40 × NA 1.3 objective. All fluorescent pictures are z-stack images consisting of 10 to 20 layers with a 0.5–0.7 μm step size. Heights for z-stack were identified manually by imaging DAPI on area of interest. Each z plane was imaged across 3–4 channels. Processing and quantification of images was carried out using the open-source software FIJI ImageJ version 2.0.0. Nikon images were denoised and deconvolved using the Nikon NIS-Elements AR 5.40.01 software. For quantification, a minimum of two representative regions of interest of at least four samples were selected. We determined *p* values as follows: * ≤ 0.05, ** ≤ 0.01, *** ≤ 0.001. Analysis and visualization were carried out using open-source software R version 4.0.3.

## Results

### Tissue processing and droplet-based single-nucleus RNA-sequencing

To investigate transcriptomic changes of heterogeneous cell populations involved in MS pathology, as well as susceptibility of different CNS regions, we performed a comprehensive single-nucleus and spatial validation analysis of 19 leukocortical, 10 cerebellar and 12 spinal cord tissue blocks obtained from MS and corresponding control subjects. Specifically, we performed IHC combined with single-molecule fluorescence in situ hybridization (smFISH) assays on all tissue samples and droplet-based snRNA-seq on 6 cerebellar and 6 spinal cord samples and profiled nuclear transcriptomes together with 8 leukocortical samples [48] (Suppl. Table 1, Online Resources). We selected MS samples that showed demyelination and assessed inflammatory lesion activity using MOG, CD45, IBA1, CD68 and CD3 IHC (Suppl. Fig. 1, Online Resources). Except three samples, which were classified as chronic-inactive, all samples harbored chronic-active lesions with partial evidence for ongoing active demyelination at lesion rims and neighboring lesion core areas. In addition, for snRNA-seq we selected samples based on RNA integrity, including only samples with an RNA integrity number (RIN) ≥ 6.5 (Suppl. Table 1, Online Resources). Samples were dissociated in lysis buffer and nuclei were isolated through ultracentrifugation in sucrose solution, followed by 10X genomics barcoding, cDNA library construction and sequencing (see “Methods”).

### Data integration and unsupervised clustering

**Fig. 1:**
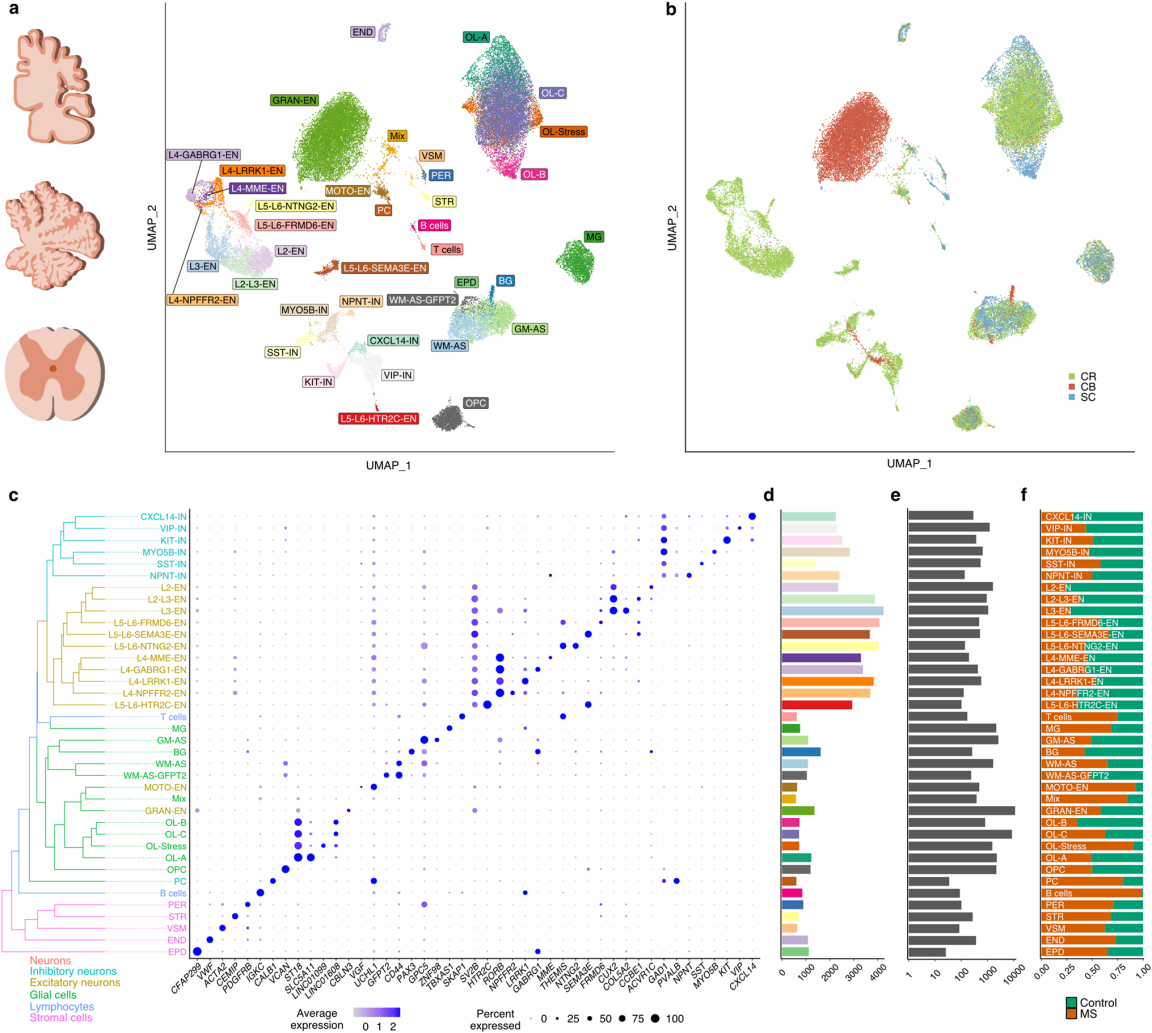
Transcriptomic integration and cell type-specific characterization of cross-regional snRNA-seq profiles from MS and control CNS tissues. **a** UMAP plot depicting 45,183 nuclei (Control, *n* = 19,718; MS, *n* = 25,465) partitioned into 38 cell type-specific clusters. Nuclei were obtained from cerebrum (leukocortical tissue: MS, *n* = 4; Control, *n* = 4), cerebellum (MS, *n* = 3; Control, *n* = 3) and spinal cord (MS, *n* = 3; Control = 3). *EN* excitatory neuron, *L* layer, *GRAN-EN* granular cell, *IN* inhibitory neuron, *PC* Purkinje cell, *PER* pericyte, *STR* stromal cell, *EPD* ependymal cell, *VSM* Vascular smooth muscle cell, *END* endothelial cells, *MG* microglia, *BG* Bergmann glia, *AS* astrocytes, *GM* gray matter, *WM* white matter, *OPC* oligodendrocyte precursor cell, *OL* oligodendrocyte. **b** Nuclei distribution based on regional origin. CR, cerebrum; CB, cerebellum; SC, spinal cord. **c** Expression and specificity of marker genes used for cell type identification. Phylogenetic tree of cell types is constructed based on the average gene expression of normalized and scaled data. **d** Median number of genes detected in each cluster. Of note, the color code of the displayed bars refers to the color code of cell type clusters in (**a**). **e** Total number of cells per cluster. **f** Proportion of control and MS cells per cluster

After quality control of each region (Suppl. Figs. 2 and 3), we obtained transcriptomic profiles of 45,183 nuclei, which were then integrated for comparative analysis using the harmony algorithm [27]. Following integration, clustering analysis and cell type classification was performed, identifying 38 distinct cellular subtypes based on marker gene expression (Fig. 1a–c and Suppl. Fig. 3, Suppl. Table 2, Online Resources, interactive web browser: https://ms-cross-regional.cells.ucsc.edu). While most clusters representing glial cell types showed a uniform regional distribution, we also identified several region-specific cell type clusters. For example, cerebellar Bergmann glia, granular and Purkinje cells, as well as spinal cord-derived ependymal cells formed distinct populations (Fig. 1a, b). In addition, we found a small cluster of nuclear profiles showing a Schwann cell gene expression signature, most likely derived from spinal roots attached to the spinal cord tissues sequenced (Suppl. Fig. 3 k, l, Online Resources). In fact, we could even distinguish myelinating from non-myelinating Schwann cells based on gene expression. Further, we found that a subtype of excitatory neurons from spinal cord and leukocortical tissues formed one cluster, which we annotated ‘motor neurons’ and was characterized by specific expression of *UCHL1* but not *CALB1*, a specific marker gene for Purkinje cells (Fig. 1c). Unsupervised hierarchical clustering revealed that related cell types grouped together highlighting conserved transcriptomic traits, e.g., between inhibitory vs. excitatory neurons and stromal/endothelial vs. glial cell types (Fig. 1c). Of note, through inter-cluster analysis of the integrated data set (Fig. 1d–f), we confirmed our previous finding that upper cortical layer excitatory neurons showed the strongest drop-out in MS relative to deep layer excitatory and other inhibitory neurons (Fig. 1f) [48]. In addition, we found distinct cell type-specific patterns of transcription factor (TF) activity between major cell types integrating transcriptomic profiles from MS and control samples across all regions (Suppl. Fig. 4, Online Resources). For example, NR2F2, a nuclear receptor activated by retinoic acid and playing a role in remyelination pathways [21], and SOX10 were specific for the oligodendrocyte lineage (Suppl. Fig. 4d, Online Resources).

### Overlapping transcriptomic features across neuroglial cell types in MS pathology

**Fig. 2:**
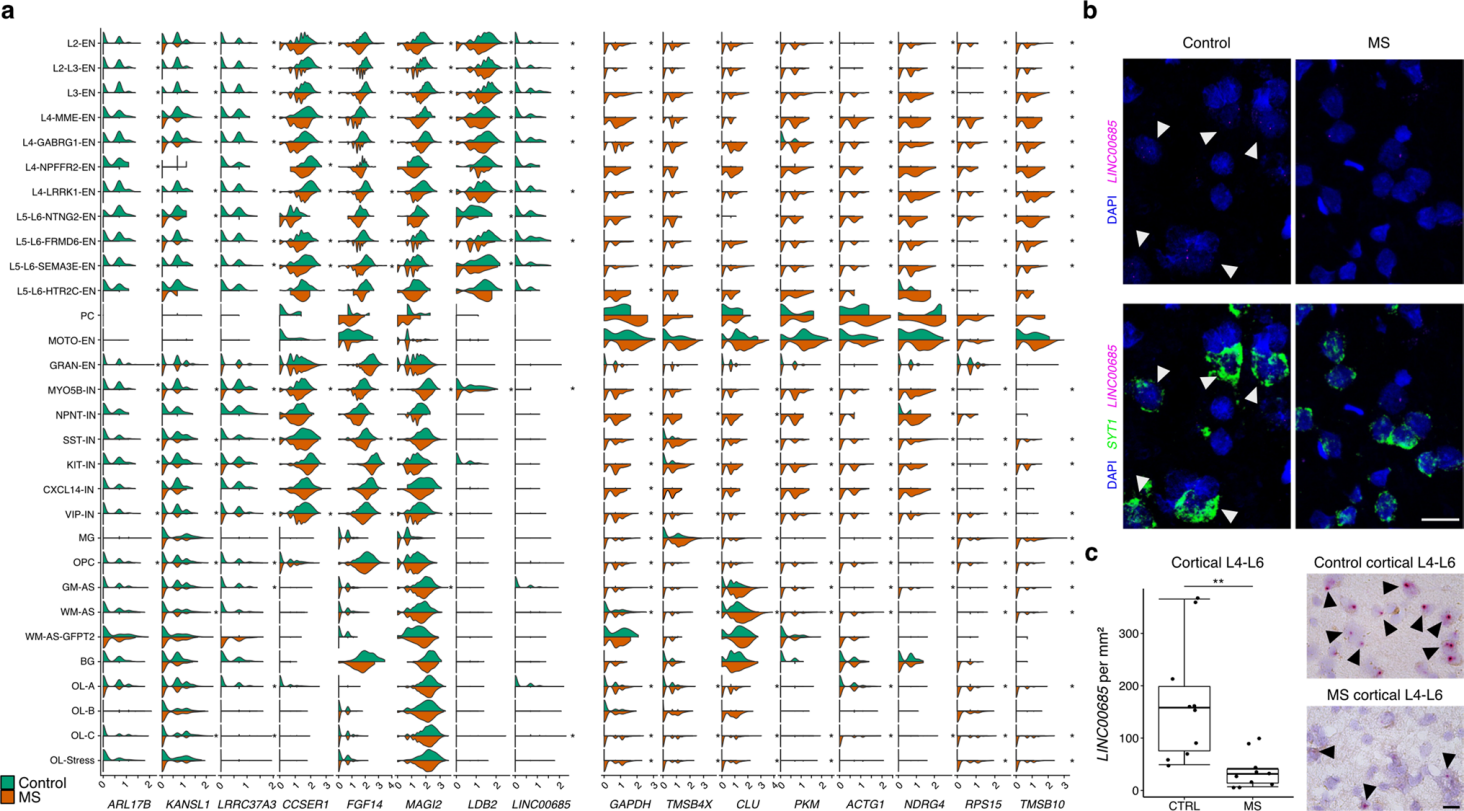
Neuroglial subtypes show shared dysregulated transcriptomic patterns in MS. **a** Violin plots showing top differentially expressed genes in MS across neuroglial subtypes. Genes are ordered based on the frequency in how many cell types they are dysregulated. Cell type populations, in which a gene is dysregulated, are marked with an asterisk (*p* < 0.05). **b** RNA in situ validation of *LINC00685* and *SYT1*. White arrowheads mark *LINC00685-*expressing cells. Scale bar indicates 20 µm. **c** RNA in situ validation of *LINC00685* between controls and MS in deep gray matter layers. Boxplots show total number of *LINC00685* signals per mm^2^. Scale bar indicates 20 µm. Data were tested for normality distribution with Shapiro–Wilk test. Then, Wilcoxon–Mann–Whitney test was used for statistical analysis, **p < 0.01

To investigate global effects of MS across CNS regions, we performed differential gene expression analysis for distinct cell types and ranked the resulting genes based on the frequency of their occurrence across different cell types. Despite a strong level of diversity within neuron subtypes (Fig. 1a, b), we observed a highly overlapping transcriptomic response pattern in MS when investigating overlaps in dysregulated gene patterns (Fig. 2a, Suppl. Table 3). *ARL17B* was the top downregulated transcript among neuroglial subtypes followed by *KANSL1*, which is associated with Koolen–De Vries syndrome and severe oxidative stress [26, 31]. Interestingly, both of these genes localize to the 17q.21.31 locus, a genomic region of high linkage disequilibrium known to contribute to other neurological diseases such as Parkinson’s disease and supranuclear palsy [5, 42]. Among other downregulated neuroglial transcripts were several long non-coding RNAs such as *LINC00685*, which is expressed in various cell types [10] with yet unknown function. We validated the spatial expression of *LINC00685* and found a strong reduction in MS deep cortical layers relative to controls (Fig. 2b, c). Transcripts strongly upregulated across all cell types, such as *GAPDH, CLU* and *PKM,* were associated with cell stress and metabolic exhaustion in MS, which is in line with previous findings [48] (Fig. 2a).

By integrating snRNA-seq data sets from three major CNS lesion sites, we observed a strong level of transcriptomic diversity among neuronal cell types in the normal CNS. Differential gene expression analysis, however, revealed a substantial overlap in dysregulated genes, resulting in shared transcriptomic response patterns in MS across various neuroglial cell types.

### Cross-regional diversity of control and MS astrocytes

**Fig. 3:**
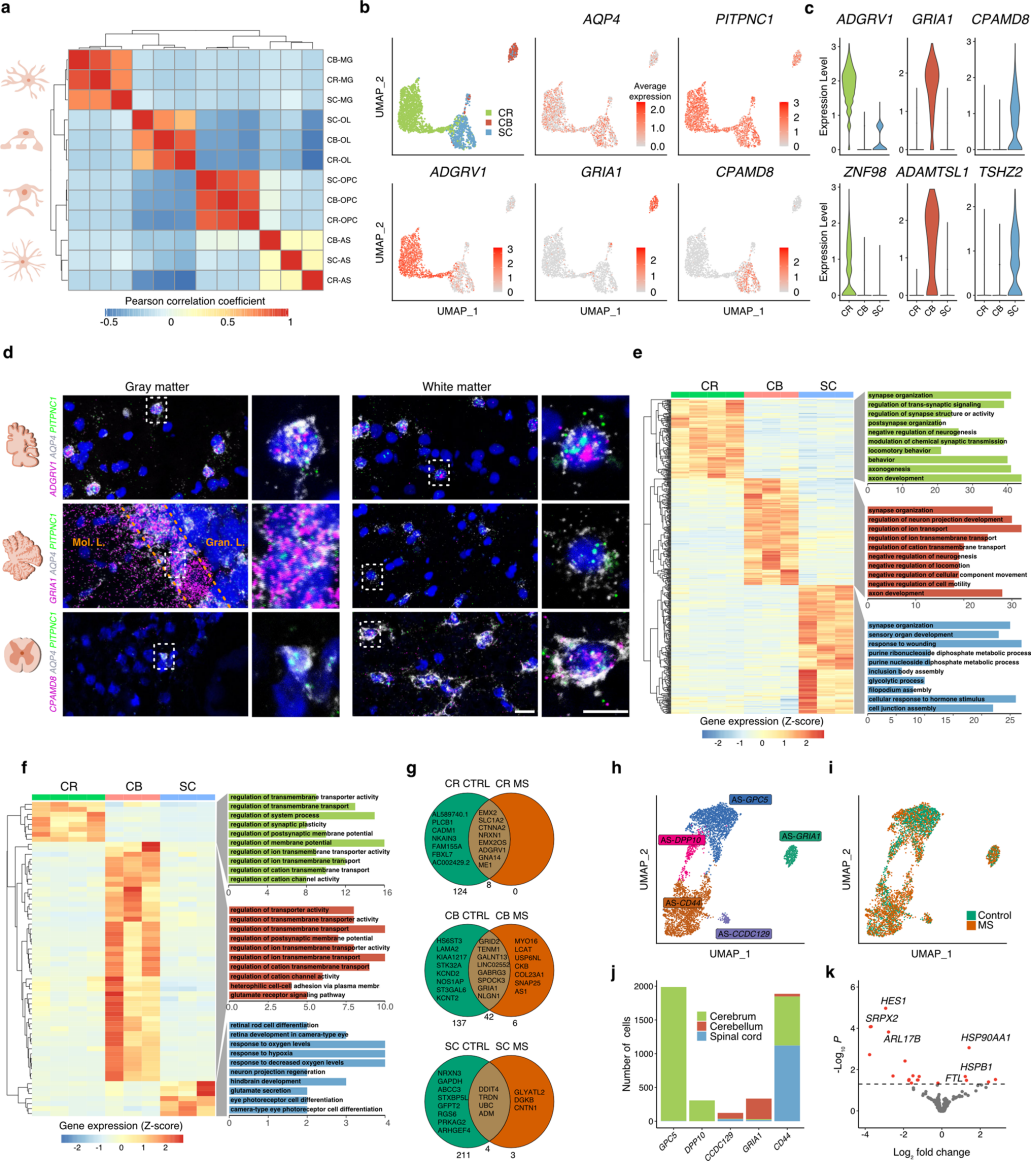
Regional diversity of homeostatic and reactive astrocytes. **a** Pearson correlation of averaged expression profiles of control glial cells from cerebrum (CR), cerebellum (CB) and spinal cord (SC). **b, c** Expression and specificity of region-specific marker genes in control astrocytes visualized by UMAP (**b**) and violin plots (**c**). Scale bars in **b** represent average expression. **d** RNA in situ validation of pan-regional astrocyte marker gene *PITPNC1* as well as region-specific astrocyte marker genes *GRIA1*, *ADGRV1*, *CPAMD8* in cerebrum, cerebellum and spinal cord, respectively. Scale bars indicate 20 µm (full size image) and 10 µm (zoom in). *Mol. L.* molecular layer, *Gran. L.* granular layer. **e** Normalized and scaled expression of DEGs (adjusted *p* < 0.05) in control astrocytes across regions and corresponding GO terms. *DEGs* differentially expressed genes, *GO* gene ontology. **f** Normalized and scaled expression of DEGs (adjusted *p* < 0.05) in MS astrocytes across regions and corresponding GO terms. **g** Venn diagram showing overlap of region-specific astrocyte genes between control and MS tissues. Bottom numbers indicate sum of genes associated with either control, MS or both. **h** Astrocyte (AS) subtypes present in cerebrum (CR), cerebellum (CB) and spinal cord (SC) tissues of control and MS tissues. **i** AS subtypes colored by their origin from control or MS samples. **j** AS subtype fractions by CNS regions. **k** Volcano plot showing differentially expressed genes between control and MS samples. DEGs are shown for *CD44*^+^ AS subtypes originating from spinal cord. Significantly differentially expressed genes are colored in red (adjusted *p* < 0.05)

We next focused on glial cell types and their subtype-specific response patterns in MS. First, to exclude disease-specific effects on AP regional heterogeneity, we only analyzed nuclei from control tissue samples and observed the strongest level of cross-regional heterogeneity among astrocytes (Fig. 3a) [3], consistent with the idea that astrocyte-driven CNS patterning may confer long term region-restricted diversity [44].

Within astrocytes, we identified *PITPNC1,* encoding a cytoplasmic phosphatidylinositol transfer protein, showing robust expression in astrocytes across all three CNS regions (Fig. 3b and d, Suppl. Fig. 5a). Next, we searched for region-specific astrocyte marker genes based on differential gene expression (DGE) analysis and identified *ADGRV1* in leukocortical, *GRIA1* in cerebellar and *CPAMD8* in spinal cord astrocytes as the top region-specific astrocyte marker genes (Fig. 3b and c, Suppl. Table 4). By smFISH, we found that *GRIA1* expression was specific to Bergmann glia in the cerebellum, and *CPAMD8*, encoding a protease inhibitor, was specific to white matter spinal cord astrocytes. *ADGRV1,* however, showed high expression levels both in gray and white matter leukocortical astrocytes (Fig. 3d, Suppl. Fig. 5a).

To gain more functional insights into astrocyte subtype diversity, we performed gene ontology (GO) term enrichment analysis and identified astrocyte region-specific biological processes that matched well their AP region-dependent tasks in support of neuron subtype function, highlighting a strong degree of functional diversity among astrocyte subtypes (Fig. 3e, Suppl. Table 5) [4, 19]. In general, pathways related to synapse organization were among the top astrocyte GO terms in all three regions. Cerebral astrocytes were enriched for pathways related to the regulation of trans-synaptic signaling and neurotransmission. Conversely, cerebellar astrocytes, which mainly comprise Bergmann glia, were enriched for neuron projection development and ion, specifically cation, transport. In spinal cord astrocytes, we identified several genes contributing to metabolic processes such as anaerobic glycolysis and catabolic processes indicating specific processes that support spinal neurons (Fig. 3e).

To further elucidate functional features of astrocyte subtypes, we focused on cell–cell interaction between Bergmann glia and Purkinje cell, as a close spatial relationship between both cell types is known. Ligand–receptor analysis identified 97 ligand–receptor interactions (Suppl. Fig. 5b, Suppl. Table 6, Online Resources) [13]. Specifically, we found genes, which are known to play critical roles in Purkinje cell development and axonal growth. For example, we identified *PTCH1*, encoding an important receptor involved in sonic hedgehog signaling in Bergmann glia, and genes encoding important cell adhesion proteins like *L1CAM* and *CHL1*, which play roles in axonogenesis in the cerebellum, based on ligand–receptor analysis (Suppl. Fig. 5b, Online Resources) [1, 23].

Since we observed high levels of cross-regional diversity between control astrocyte subtypes, we next calculated inter-regional differences in MS astrocytes. Notably, we observed a sharp decline in the number of region-specific astrocyte genes in MS (Fig. 3f, g, Suppl. Table 7, 8, Online Resources), with a small set of homeostatic core genes shared between control and MS samples and remaining in each region (Fig. 3g). Also, when performing ligand–receptor analysis for Bergmann glia and Purkinje cells from MS samples only we observed a decrease in ligand–receptor pairs relative to controls (97 vs. 73). Specifically, *L1CAM* (encoding the cell adhesion protein L1) signaling from Purkinje cells became absent in MS with potentially important implications for axon repair and neuronal self-defense in the context of neuroinflammation (Suppl. Fig. 5b, Online Resources) [38]. Further, consistent with the decreased number of region-specific astrocyte genes in MS, we observed a decrease in region-specific TF activity in MS astrocytes (Suppl. Fig. 6a, b, Online Resources).

In summary, we noted that control astrocytes represent a highly heterogeneous glial cell type across the CNS with regional heterogeneity becoming more obscure when focusing on MS astrocytes.

### Subregional white and gray matter diversity of homeostatic and reactive astrocytes

Next, we focused on subregional astrocyte responses in MS between gray and white matter areas. First, we pooled snRNA-seq profiles from control and MS astrocytes, which enabled us to analyze homeostatic cells that are derived from control and NAWM MS tissue areas as well as reactive subtypes that mainly derived from MS lesion areas [46]. This approach allowed the identification of 5 distinct astrocyte populations (Fig. 3h–j, Suppl. Fig. 6c, Suppl. Table 9, Online Resources). One subtype (AS-*GPC5*) was characterized by high expression of known cortical gray matter astrocytes marker genes, such as *GPC5* and *SLC1A2* [48]. A second cluster (AS-*GRIA1*) was enriched for *PAX3*, *ADAMTSL1* and *GRIA1*, the latter one a known marker associated with cerebellar Bergmann glia function [45]. A third cluster (AS-*CD44*) showed high expression levels of *CPAMD8* and *CD44*, the latter one a known marker for subcortical white matter astrocytes [7, 48]. Further, we identified two additional populations with high expression of *SLC17A7/DPP10* (AS-*DPP10*) and *CCDC219/PLK5* (AS-*CCDC219*) (Fig. 3h and j). The AS-*CD44* population mainly comprised astrocyte profiles from cerebral (subcortical) and spinal cord (white matter tracts) origin; conversely, AS-*GPC5*/AS-*DPP10* profiles were mainly derived from cerebral (cortex) and AS-*GRIA1/*AS-*CCDC219* from cerebellar tissues (Fig. 3j). In addition, using differential TF activity analysis, we found evidence for an enhanced TF activity of SOX11 in AS-*GPC5* and ATF2 in AS-*CD44* astrocytes suggesting differential TF activity between gray and white matter astrocytes (Suppl. Fig. 6d, Online Resources).

To gain more insight into subregional gene alterations in MS astrocytes, we performed differential gene expression analysis and focused on white matter AS-*CD44* astrocytes. We identified 16 differentially expressed genes (DEGs) in subcortical (cerebral) versus 21 DEGs in spinal cord MS-specific white matter astrocytes (Fig. 3k, Suppl. Fig. 6e, Suppl. Table 10, Online Resources)*.* Specifically, we found that *ARL17B* (see above), *HES1* (encoding a basic helix-loop-helix transcription factor) and *SRPX2* (encoding a protein relevant for glutamatergic synapse formation) were selectively downregulated in spinal AS-*CD44* cells in MS; conversely, known stress marker genes such as *HSP90AA1*, *HSPB1* and *FTL* appeared to be upregulated in spinal MS astrocytes (Fig. 3k).

Combining control with MS astrocytes allowed identification of subregional transcriptomic signatures pointing towards distinct gray and white matter subtypes across the CNS. In particular, we found that white matter astrocyte signatures overlap between cerebral and spinal regions with specific patterns of reactivity in MS.

### Regional and subregional diversity of homeostatic and reactive oligodendrocytes

**Fig. 4:**
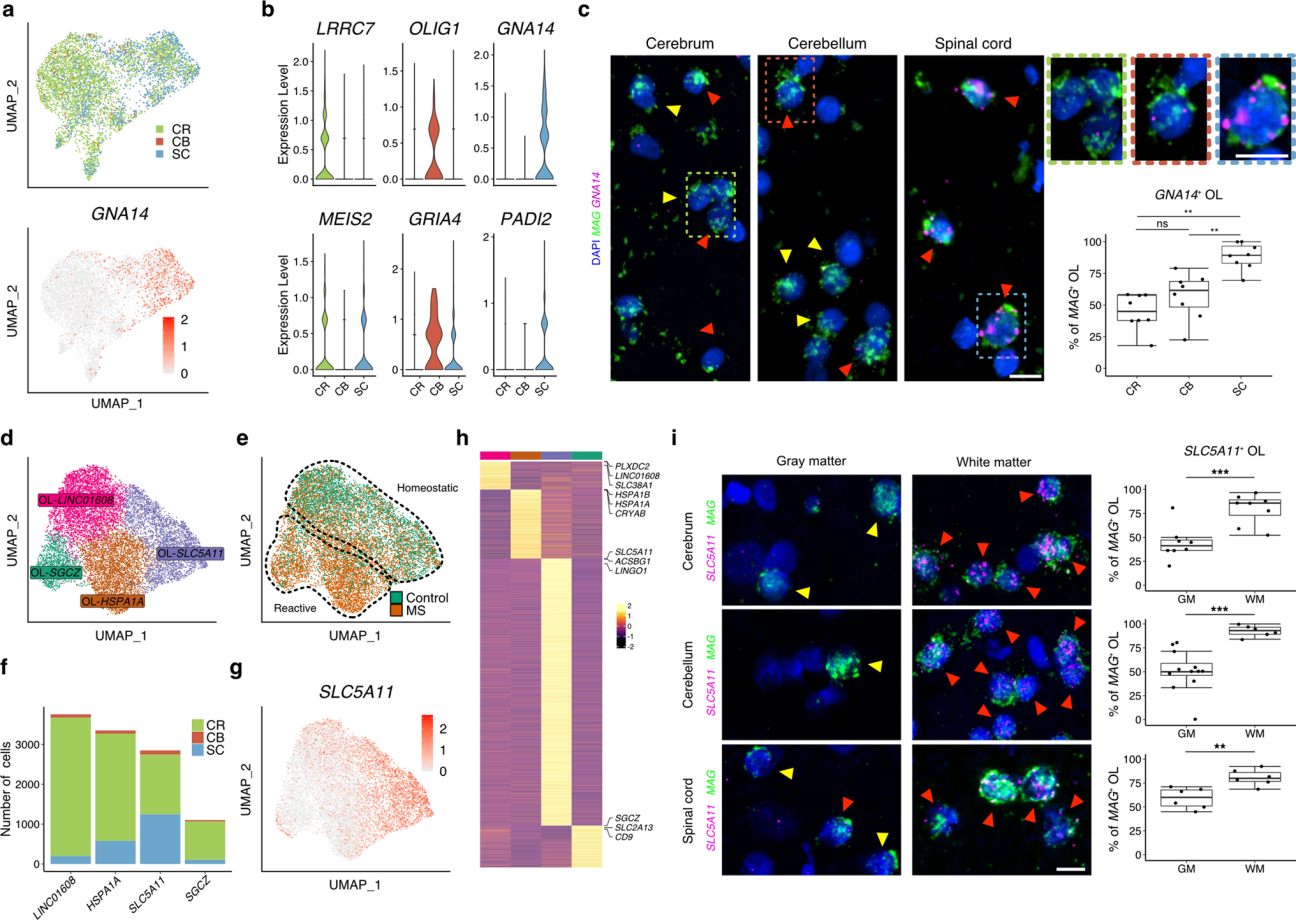
Regional diversity of homeostatic and reactive oligodendrocytes. **a, b** Expression and specificity of region-specific marker genes in control oligodendrocytes visualized by UMAP (**a**) and violin plots (**b**). Scale bar in (**a**) represents average expression. **c** RNA in situ validation of spinal white matter-specific oligodendrocyte marker gene *GNA14* across all three CNS regions. Yellow arrowheads mark *MAG*-expressing (*MAG*^+^) OLs, whereas red arrowheads mark *GNA14/MAG*-expressing (*GNA14*^+^*/MAG*^+^*)* OLs. Scale bars indicate 10 µm (full size image) and 10 µm (zoom in). Boxplots show *GNA14*^+^ OLs (*GNA14*^+^/*MAG*^+^) in percentage of all OLs (*MAG*-expressing cells, *MAG*^+^). Data were tested for normality distribution with Shapiro–Wilk test. Then, Kruskal–Wallis Test was used for statistical analysis. For comparison of each group, pairwise Wilcoxon test with Bonferroni correction was performed, *p < 0.05, **p < 0.01, ***p < 0.001. **d** Oligodendrocyte (OL) subtypes present in cerebrum (CR), cerebellum (CB) and spinal cord (SC) tissues of control and MS tissues. **e** OL subtypes colored by their origin from control or MS samples. Of note, OL clustering showed a separation into homeostatic and reactive subtypes. **f** OL subtype fractions by CNS regions. **g** UMAP plot showing specific expression of the OL white matter subtype marker gene *SLC5A11*. **h** Heatmap showing expression of subtype-specific gene clusters. For each subtype, three representative genes are listed in the heatmap. Grouping color bar matches colors for subtypes in (**d**). **i** RNA in situ validation of white matter subregional OL marker gene *SLC5A11*. Yellow arrowheads mark *MAG*-expressing (*MAG*^+^) OLs, whereas red arrowheads mark *SLC5A11/MAG*-expressing (*SLC5A11*^+^*/MAG*^+^*)* OLs. Staining was carried out for all three CNS regions; cerebellar gray matter image was taken in molecular layer; gray matter quantification was carried out in both granular and molecular layers. Scale bar indicates 10 µm. Boxplots show *SLC5A11*^+^ OLs (*SLC5A11*^+^/*MAG*^+^) in percentage of all OLs (*MAG*-expressing cells, *MAG*^+^). Data were tested for normality distribution with Shapiro–Wilk test. Then, Welch's *t* test was used for statistical analysis, *p < 0.05, **p < 0.01, ***p < 0.001

Next, we focused on oligodendrocytes—the natural cellular target in MS with a high level of stress response including iron dysregulation and antigen presentation in MS lesions [22, 24, 48]. In comparison to astrocytes, we found only small differences between oligodendrocytes obtained from control samples across all three CNS regions (Fig. 4a). Specifically, we identified *LRRC7* as a cerebrum-specific oligodendrocyte marker gene, *OLIG1* to be enriched in cerebellar oligodendrocytes and *GNA14*, encoding for a member of the Gq alpha subunit family, as a specific marker for spinal cord oligodendrocytes (Fig. 4b, Suppl. Table 11, Online Resources). Using smFISH, we confirmed the specific expression of *GNA14* in spinal white matter oligodendrocytes (Fig. 4c). Although we found a certain degree of *GNA14* expression in other regions, *GNA14* expression was highest in *MAG*-expressing oligodendrocytes of spinal white matter tracts. Further, we found only little cross-regional AP diversity between control microglia and oligodendrocyte progenitor cells (Suppl. Fig. 7, Suppl. Table 12, 13, Online Resources).

To gain insight into subregional diversification of oligodendrocytes and their changes in MS, we then pooled nuclear profiles from control and MS samples and identified two homeostatic oligodendrocyte subtypes (mix of nuclei from control and MS samples) characterized by specific expression of *LINC01608* and *SLC5A11,* as well as two reactive subtypes enriched for nuclei from MS samples and characterized by expression of *HSPA1A* and *SGCZ* (Fig. 4d, e, Suppl. Fig. 8d, Suppl. Table 14, Online Resources). The *SLC5A11*-enriched homeostatic subtype was composed of a mix of nuclei from cerebral and spinal cord samples (Fig. 4f, g), suggesting white matter origin like AS-*CD44* astrocytes (Fig. 3j). TF activity analysis identified TFs such as SOX10 and NR2F2 [32] specific for *SLC5A11*-expressing and PAX6 [12] for *LINC01608*-expressing oligodendrocytes (Suppl. Fig. 8a, Online Resources). As the two reactive subtypes were characterized by high expression of genes associated with cell stress and metabolic exhaustion, we focused on the two homeostatic clusters that comprised oligodendrocytes from both control and MS tissues to specifically examine changes in MS cells “at risk”, which could still benefit from MS therapies. Of note, the OL-*SLC5A11* subtype was characterized by a specific expression of *ACSBG1* and *LINGO1,* the latter one encoding a protein known to inhibit OPC differentiation and therefore might inhibit remyelination in MS lesions [39] (Fig. 4h, Suppl. Fig. 8b, c, Online Resources). To investigate the spatial distribution of OL-*SLC5A11* cells, we applied smFISH, and found that *SLC5A11* expression was strongly associated with white matter tract oligodendrocytes across all three CNS regions (Fig. 4i, Suppl. Fig. 8e, Online Resources). Conversely, we confirmed that OL-*LINC01608* cells were found in both gray and white matter compartments across the regions (Suppl. Fig. 8f, Online Resources). To validate specificity of our approach, we compared subregional oligodendrocyte marker gene expression with data from a previously published data set obtained from human subcortical tissues (controls and MS) [22]. In comparison with the Jäkel et al. study and when focusing on *SLC5A11* and *LINC01608* expression, we could confirm subtype diversification with gene expression enriched in distinct oligodendrocyte clusters as suggested (Suppl. Fig. 8 g, Online Resources). Conversely, we showed that expression of *OPALIN*, a known oligodendrocyte marker gene, was associated with OL-*LINC01608* and OL-*SGCZ* clusters but not white matter oligodendrocytes (Fig. 4f, Suppl. Fig. 8 h, Online Resources) [22]. Further, in our data set we found only weak expression of *CD74*, a marker gene linked to antigen presentation in reactive oligodendrocytes, in the MS-specific OL-*HSPA1A* population (Suppl. Fig. 8 h, Online Resources) [22, 48].

In summary, these results demonstrate cross-regional and, more specifically, subregional diversification of homeostatic oligodendrocytes in control and MS tissues highlighting gray and white matter areas distinguished by specific marker genes.

### MS-associated white matter oligodendrocytes show a pro-myelinating signature

**Fig. 5:**
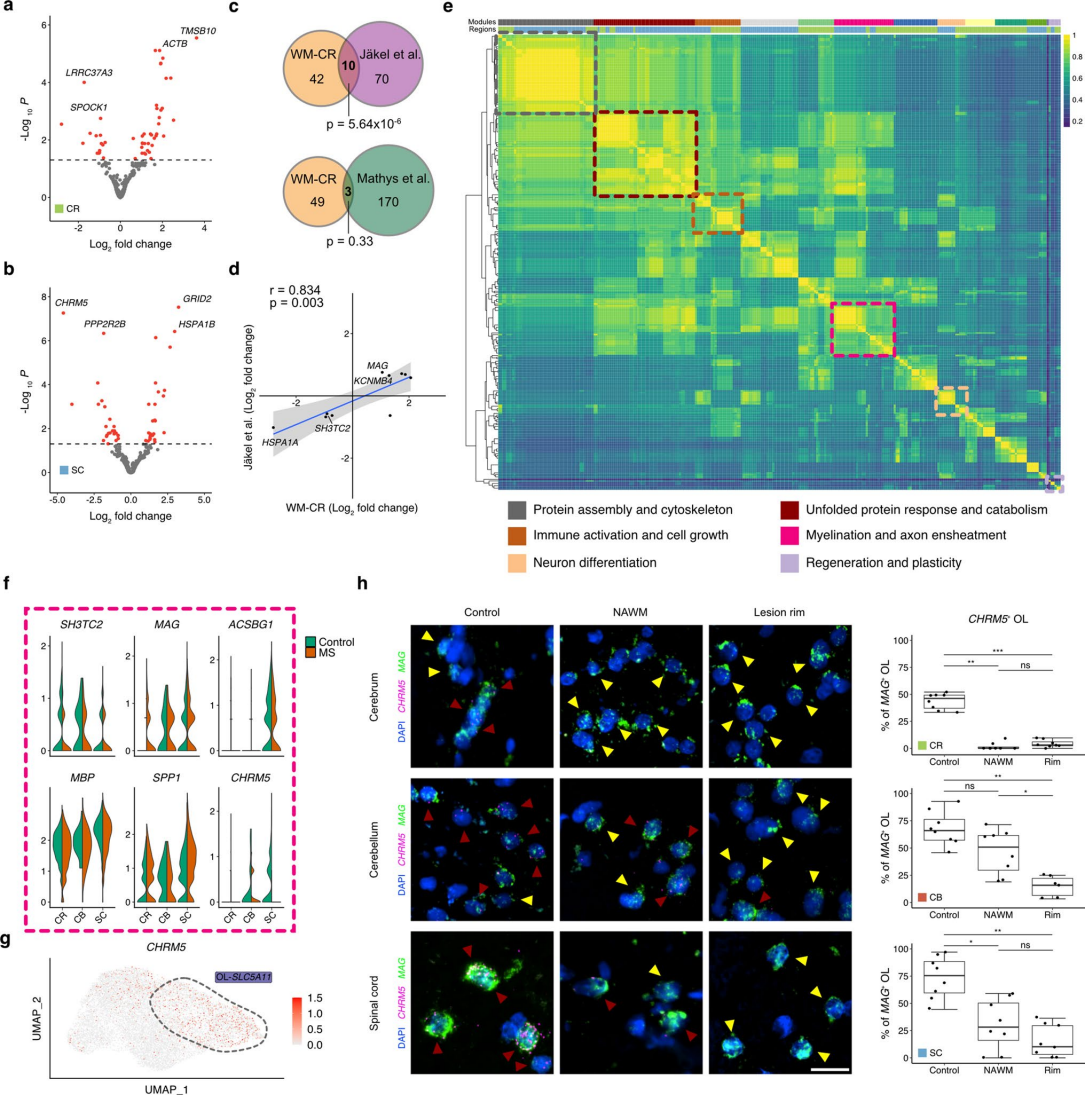
Cross-regional white matter oligodendrocytes have pro-myelinating signatures. **a**, **b** Volcano plots showing differentially expressed genes between control and MS samples. **a** DEGs are shown for *SLC5A11*^+^ OL subtypes originating from cerebrum (leukocortical). **b** DEGs are shown for *SLC5A11*^+^ OL subtypes originating from spinal cord. Significantly differentially expressed genes are colored in red (adjusted *p* < 0.05). **c** Venn diagrams showing overlap between DEGs of leukocortical (CR) *SLC5A11*^+^ OL subtype with DEGs from OL subtypes from Jäkel et al. [22] (upper panel) and from Mathys et al. [36] (lower panel). Of note, OL subtypes showing high expression levels of *SLC5A11* were used for comparison with Jäkel et al. For comparison with Mathys et al., only one OL subtype was available, which was used for comparison. **d** Pearson correlation analysis of log2 fold changes of overlapping DEGs between CR *SLC5A11*^+^ OL and Jäkel et al. **e** Semantic similarity analysis of enriched pathways. Gene clusters corresponding to GO terms identified for genes in **a** and **b** were used as input for semantic similarity correlation analysis. The heatmap shows calculated correlations between each identified GO term gene cluster. Hierarchical clustering of GO terms revealed functional modules indicated by the upper color bar between both subcortical and spinal OLs as indicated by lower color bar (cerebrum = green, spinal cord = blue). Note six clusters showed overlap between the two CNS regions. GO terms identified with adjusted *p* < 0.1 were included. **f** Violin plots showing dysregulated genes in MS which contributed to the pink module in (**c**). **g** UMAP plot showing the expression of *CHRM5* in white matter OL-*SLC5A11* subtype. **h** RNA in situ validation of MS lesion-associated downregulation of *CHRM5*. Yellow arrowheads mark *MAG*-expressing (*MAG*^+^) OLs, whereas red arrowheads mark *CHRM5/MAG*-expressing (*CHRM5*^+^*/MAG*^+^*)* OLs. Staining was carried out for all three CNS regions. Scale bar indicates 10 µm. Boxplots show *CHRM5*^+^ OLs (*CHRM5*^+^/*MAG*^+^) in percentage of all OLs (*MAG*-expressing cells, *MAG*^+^). Data were tested for normality distribution with Shapiro–Wilk test. Then, Kruskal–Wallis Test was used for statistical analysis. For comparison of each group, pairwise Wilcoxon test with Bonferroni correction was performed, *p < 0.05, **p < 0.01, ***p < 0.001

To gain additional insight into *SLC5A11*-expressing white matter subcortical and spinal oligodendrocytes in response to chronic inflammatory demyelination, we performed DGE analysis and identified 52 dysregulated genes for each population (Fig. 5a, b). To test if these genes were specifically enriched in MS, we next focused on the DEGs from subcortical tissues (WM-CR) and compared these genes with the DEGs derived from subcortical oligodendrocyte populations enriched for *SLC5A11* expression based on a previous study on MS snRNA-seq by Jäkel et al. (Suppl. Fig. 8g, Online Resources) [22]. Of note, we observed a strong overlap in OL-*SLC5A11* DEGs between both studies, whereas the overlap was not significant when comparing to another cerebral snRNA-seq dataset obtained from control and Alzheimer’s disease tissues (Fig. 5c) [36]. Furthermore, we found that shared DEGs between both MS snRNA-seq studies (Jäkel et al. and ours) were dysregulated in the same direction with *MAG*/*KCNMB4* upregulated and *SH3TC2/HSPA1A* downregulated in MS OL-*SLC5A11* cells (Fig. 5d). The latter findings point towards a potential repair function of these cells in MS as myelin (*MAG*) and potassium channel genes (*KCNMB4*) appeared to be upregulated.

To further explore the overlap of stress responses induced by MS in the OL-*SLC5A11* white matter subtype, we performed GO term enrichment with consecutive semantic similarity analysis and found a substantial level of overlap resulting in 6 convergent functional modules between both white matter subtypes. While the first three modules were enriched for pathways associated with cell stress (“protein assembly and cytoskeleton”, “unfolded protein response and catabolism”, “immune activation and cell growth”), others were related to myelination, neuron differentiation and plasticity (Fig. 5e, Suppl. Table 15, Online Resources). As myelination and remyelination are key pathways in MS pathobiology, we then focused on genes which contributed to the “myelination and axon ensheathment” module. Based on DGE and GO term enrichment analysis, we then identified *CHRM5*, encoding the muscarinic acetylcholine receptor M_5_, to be substantially downregulated in spinal cord white matter MS oligodendrocytes (Fig. 5a, e–g), whereas myelin-associated transcripts like *MAG* and *MBP* appeared to be upregulated in MS. To study subregion-specificity of *CHRM5*, we used smFISH of control and MS tissues comparing white matter areas of all three CNS regions. Notably, as compared to control tissue, we found a strong downregulation of *CHRM5* in *MAG*-expressing oligodendrocytes in both normal-appearing and lesion rim areas in a gradual pattern across all three regional white matter tracts (Fig. 5h). Other OL-*SLC5A11* white matter genes included in the “myelination and axon ensheathment” module were *SH3TC2* and *ACSBG1*, both genes known to encode proteins involved in myelination (Fig. 5f). For example, *SH3TC2* has been described to be expressed in Schwann cells and plays a critical role in the formation of the node of Ranvier [2]. Mutations in this gene cause Charcot–Marie–Tooth disease type 4C, a hereditary motor sensory neuropathy [35]. *ACSBG1* encodes for a protein with acyl-CoA synthetase activity and is involved in long-chain fatty acid metabolism and, therefore, linked to the formation of myelin [49].

Collectively, these findings point towards a cross-regional white matter tract signature attributed to oligodendrocytes, which become activated under inflammatory-demyelinating conditions and might be linked to myelination and repair pathways.

### Overlapping oligodendrocyte transcriptomic response during MS lesion progression

As for the astrocytes, we found a disease-driven convergence of TF activity in oligodendrocytes when comparing subcortical with spinal white matter tracts (Suppl. Fig. 9a–c, Online Resources). Further, when considering the inflammatory stage of subcortical MS lesions [48] (Suppl. Fig. 1, Suppl. Table 1, Online Resources), we were able to subcluster *SLC5A11*-expressing subcortical oligodendrocytes. By performing trajectory analysis, we observed two distinct endpoints representing early (acute/chronic-active) and late (chronic-inactive) lesion stages (Suppl. Fig. 9d, Online Resources). By computing DEGs between the endpoints of the two trajectories, we identified distinct gene patterns for each lesion stage. Testing for an overlap of these patterns between subcortical and spinal white matter oligodendrocytes obtained from MS samples, we observed overlapping expression in genes associated with early-stage inflammatory lesions in line with the enhanced inflammatory activity in the spinal cord lesions used for snRNA-seq (Suppl. Fig. 9d, Suppl. Tables 1 and 16, Online Resources).

In summary, these results suggest a convergence of cross-regional transcriptomic reactivity in MS oligodendrocytes pointing towards shared response patterns during lesion progression.

## Discussion

Despite a good control of the peripheral immune response in relapsing–remitting MS, patients still experience ongoing atrophy and worsening of symptoms related to different functional systems and anatomical areas [9, 53]. Hence, a better understanding of regionally restricted neuronal and glial subtype diversity and how these subtypes differ in their transcriptomic response to inflammatory demyelination, would help decode compartmentalized pathology in MS [46]. Further, identifying those spatial subtypes would catalyze the development of region- and cell type-specific biomarkers and help design targeted treatments to tackle specific cell types involved in progressive MS.

In this study, we generated an integrated cell type-specific transcriptomic atlas of MS pathology spanning three major CNS sites that are regularly affected by MS, including leukocortical, cerebellar and spinal cord areas. Investigating the similarity among cell types between these regions, we observed a strong level of molecular diversity between control cell types highlighting astrocyte and oligodendrocyte subtypes. We identified specific homeostatic signatures for regional astrocytes and demonstrated that their functional properties are paired with the needs of neighboring neurons in the respective regions. Specifically, we could identify functionally relevant core genes in Bergmann glia that encode for proteins relevant in ligand–receptor interaction with Purkinje cells. For example, we observed that genes encoding proteins with important functions in sonic hedgehog signaling and axonal development were linked to cerebellar astrocytes [8, 14].

Under disease conditions, however, neuron and macroglial subtypes showed a strong overlap in their transcriptomic response towards chronic inflammatory demyelination across distinct anatomical regions and specific subregions, including gray and white matter. Furthermore, we found that a core of downregulated transcripts across several neuroglial cell types was associated with a previously described highly vulnerable genomic area on chromosome 17 linked to neurological dysfunction.

Within the oligodendrocyte lineage, we identified two homeostatic and two reactive subtypes. By smFISH analysis, we could show that one homeostatic subtype was indeed specific to white matter tracts across all three CNS regions and enriched for the marker genes *SLC5A11* and *LINGO1*. For this oligodendrocyte subtype, we observed downregulation of *CHRM5* and, additionally, found an upregulation of myelin-associated transcripts. These findings might indicate an endogenous oligodendrocyte repair mechanism to promote remyelination, which becomes activated in MS white matter tracts including both lesion rim and normal-appearing areas. Of note, anti-LINGO1 antibodies and muscarinic receptor antagonists have been used in pre-clinical and clinical trials in MS patients showing partial efficacy [39, 41]. Beside other antimuscarinic therapies like benztropine [11], the first-generation H1 antagonist clemastine was proposed to have beneficial potentially pro-myelinating effects in MS patients [11, 37]. A follow-up clinical trial showed indeed improved visual function in clemastine-treated patients with optic neuritis [17]. These studies mainly focused on muscarine receptor M1 [37], and so far, did not discuss potential effects of receptor M5. However, comparing expression levels of muscarinic receptor subtypes throughout the human CNS, we found *CHRM5* to be the most abundant receptor subtype in the CNS. Although this suggests an important role of *CHRM5* in human CNS MS pathology, ultimately, more functional loss-of-function studies are needed to link the downregulation of *CHRM5* to the upregulation of myelin transcripts to provide a mechanistic link.

**Fig. 6:**
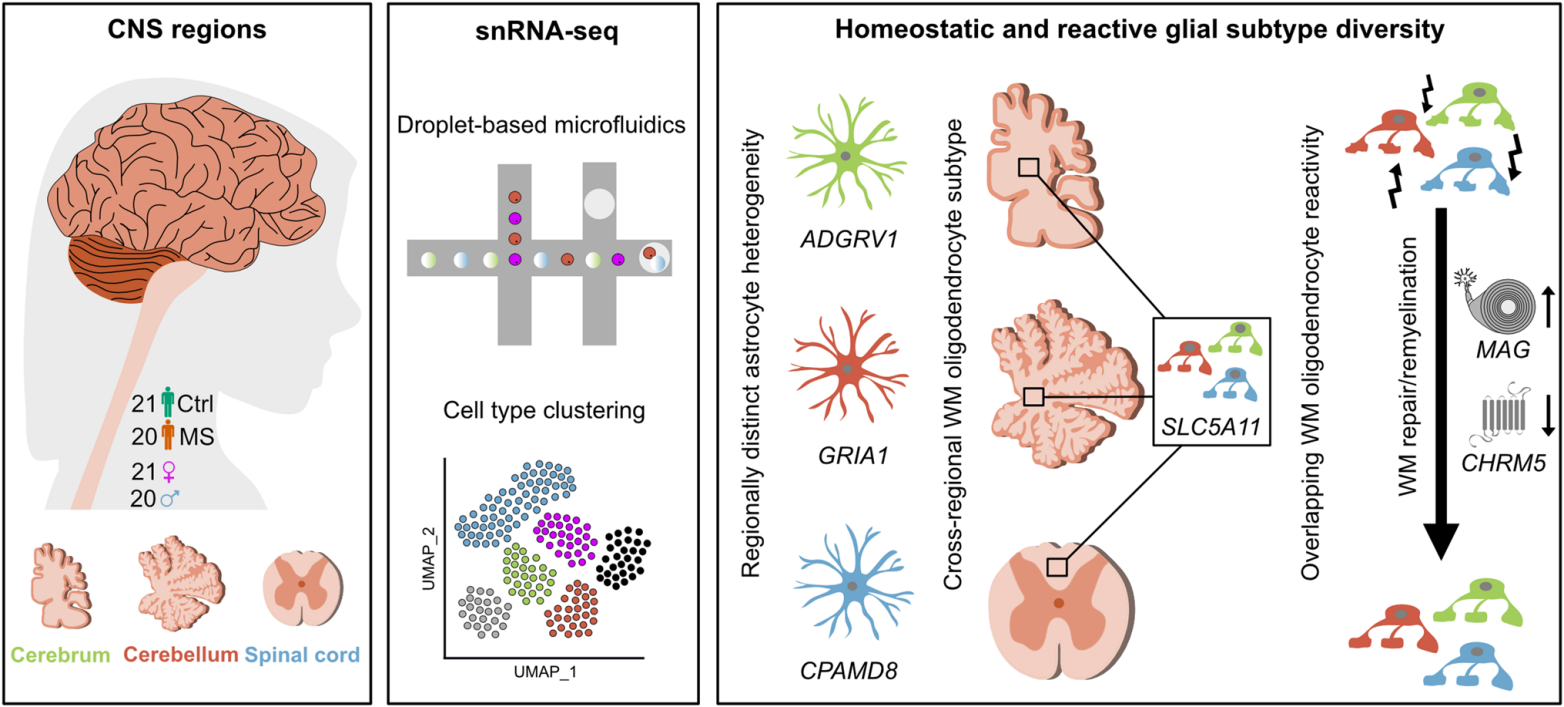
Graphical abstract highlighting key findings of the study. Illustration shows the composition of the dataset (left panel), the methodological and bioinformatic workflow (middle panel) and the results highlighting astrocyte diversity, subregional white matter oligodendrocyte diversification and overlapping transcriptomic reactivity of white matter oligodendrocytes including upregulation of the myelin-encoding gene *MAG* and downregulation of the muscarinic receptor-encoding gene *CHRM5* (right panel). *Ctrl* control, *WM* white matter

In summary, our analysis uncovered overlapping molecular patterns of cell type-specific reactivity in compartmentalized MS lesion areas with a focus on homeostatic and reactive astrocyte and oligodendrocyte subtypes. Our unbiased in silico approach was able to identify a white matter-specific oligodendrocyte subtype that was associated with previously discovered pro-myelinating therapeutic target gene expression. Further, we could demonstrate that independent of a pharmacological treatment approach, oligodendrocytes transform into a reactive state, in which pro-myelinating pathways are turned on in the context of chronic inflammatory demyelination (Fig. 6). Hence, our findings demonstrate that integrated computational workflows are highly suitable to identify common cell type-specific signatures across different CNS regions and help identify novel therapeutic targets with a broad, however, subtype-specific expression pattern.

## Supplementary Information

Below is the link to the electronic supplementary material.

Suppl. Fig. 1 Histological assessment of MS lesions. a MOG and CD45 immunohistochemistry of a representative subcortical MS lesion tissue section. An acute lesion with active demyelination with presence of foam cells (white arrowheads) in the lesion center and rim area is shown next to a chronic-active lesion with demarcated core and hypercellular lesion rim with high levels of microglia (black arrowheads). Overview images for MOG and CD45 were taken on corresponding MS lesion areas of serial sections. b MOG and CD45 immunohistochemistry for a representative cerebellar MS lesion tissue section. A chronic-active cerebellar lesion with demarcated core and hypercellular lesion rim with presence of macrophages (white arrowheads) and microglia (black arrowheads) is shown. Overview images for MOG and CD45 were taken on corresponding MS lesion areas of serial sections. c MOG and CD45 immunohistochemistry for representative spinal cord MS lesion tissue sections. Upper row shows acute spinal cord MS lesion with active demyelination and presence of macrophages (white arrowheads). Lower two rows show chronic-active lesions with demarcated lesion cores in gray and white matter areas with hypercellular lesion rims and presence of few macrophages (white arrowheads) and abundant microglia (black arrowheads). Overview images for MOG and CD45 were taken on corresponding MS lesion areas of serial sections. Scale bars indicate 500 µm on overview images and 100 µm on zoom ins. d Fluorescence immunohistochemistry for IBA1, CD68 and CD3. On the left side, NAWM with macrophages (IBA1+/CD68+, red arrowheads) and resident ramified microglia (CD68+, cyan arrowheads) is shown. On the right side, image displays a lesion core of an acute lesion. T cells (CD3+, yellow arrowheads) are shown next to tissue-infiltrating macrophages (IBA1+/CD68+, red arrowheads) coming from a nearby vessel (asterisk). Scale bar indicates 20 µm. Suppl. Fig. 2 Quality control and batch correction of snRNA-seq data. a Number of detected counts in each nucleus. Dashed line represents lower threshold to exclude nuclei for further analysis. b Number of detected genes in each nucleus. Dashed line represents lower threshold to exclude nuclei for further analysis. c Quality control metrics after filtering low quality nuclei, represented by number of detected genes and number of detected counts as well as percentage of mitochondrial genes. d, e cell distribution based on the sample of origin before (d) and after (e) batch correction. f UMAP plot of integrated object showing the distribution of cell clustering between control and MS. Suppl. Fig. 3 Cell type-specific cluster analysis of snRNA-seq data per CNS region. a, f, k UMAP plot showing cell-type-specific clusters in snRNA-seq data sets from cerebrum (leukocortical) (a), cerebellum (f) and spinal cord (i) of control and MS tissues. b, g, l Expression and cell type-specificity of marker genes in cerebrum (b), cerebellum (g) and spinal cord (l). DC, dendritic cells; MON, monocytes; vasAS, vascular astrocytes; mSCH, myelinating Schwann cells; nmSCH, non-myelinating Schwann cells; VENTRAL-NEU, ventral horn neurons; DORSAL-NEU, dorsal horn neurons (please refer to Fig. 1 for other cell type abbreviations). c, h, m Median number of genes per cluster in cerebrum (c), cerebellum (h) and spinal cord (m). d, i, n, Number of cells per cluster in cerebrum (d), cerebellum (i) and spinal cord (n). e, j, o Proportion of control and MS cells per cluster in cerebrum (e), cerebellum (j) and spinal cord (o). Suppl. Fig. 4 Cross-regional cell type-specific transcription factor activity. a UMAP plot showing MS and control nuclei clustering based on TF activity obtained from leukocortical, cerebellar and spinal cord snRNA-seq data sets. b Cell type annotation inferred from the expression profiles in Fig. 1a. c Nuclei distribution based on condition. d Patterns of TF activity across different cell types. Suppl. Fig. 5 Diversity of spinal cord astrocytes and cerebellar astrocyte functions. a RNA in situ validation confirming PITPNC1 relative to AQP4 as cross-regional pan-astrocyte marker gene and CPAMD8 as white matter-specific astrocyte marker gene in spinal cord. Scale bar indicates 200 µm. b Dotplot showing ligand–receptor analysis between cerebellar Bergmann glia and Purkinje cells. Upper x-axis annotation indicates ligand cell type (sender), whereas lower x-axis indicates receptor cell type (receiver). The left panel shows analysis for cells originating from control donors, the right panel displays analysis for cells originating from MS donors. Suppl. Fig. 6 Regional heterogeneity and transcriptomic response patterns in MS astrocytes. a Comparison of region-specific TFs in control and MS. Scale bars indicate level of TF activity. b Venn diagram showing overlap between region-specific astrocyte TF activity in control and MS. Bottom numbers indicate sum of TFs enriched in either controls, MS or both. CTRL, control. c Heatmap showing expression of subtype-specific gene clusters. For each subtype, three representative genes are listed in the heatmap. Grouping color bar matches colors for subtypes in Fig. 3h. d Diverse TF activity between five astrocytes subtypes. Top 4 altered TFs per subtype are shown. e Volcano plot showing differentially expressed genes between control and MS samples. DEGs are shown for CD44+ AS subtypes originating from cerebrum. Significantly differentially expressed genes are colored in red (adjusted p < 0.05). Suppl. Fig. 7 Regional diversity of oligodendrocyte precursor cells and microglia. a, c Cluster analysis of control oligodendrocyte precursors (OPCs) (a) and microglia (c) based on CNS region. b, d Average expression of region-specific marker genes for OPCs (b) and microglia (d). Suppl. Fig. 8 Subregional diversity of homeostatic and reactive oligodendrocytes. a Diverse TF activity between homeostatic and reactive oligodendrocyte subtypes. Top 5 altered TFs per subtype are shown. b, c, d UMAP plot showing expression of homeostatic OL-SLC5A11 white matter subtype markers LINGO1 (b) and ACSBG1 (c) as well as expression of homeostatic OL subtype marker LINC01608 (d). e RNA in situ validation of white matter subregional OL marker gene SLC5A11 as white matter specific. Scale bar indicates 150 µm. f RNA in situ validation of OL subtype marker gene LINC01608. White arrowheads mark single positive (PLP1-expressing, PLP1+) OLs, whereas red arrowheads mark double positive (LINC01608/PLP1-expressing, LINC01608+/PLP1+) OLs. Stainings are shown for cerebrum and spinal cord. Scale bar indicates 10 µm. Boxplots show LINC01608+ OLs (LINC01608+/PLP1+) in percentage of all OLs (PLP1-expressing cells, PLP1+). Data were tested for normality distribution with Shapiro–Wilk test. Then, Wilcoxon–Mann–Whitney test was used for statistical analysis, *p < 0.05. g Violin plots showing expression of OL subtype markers SLC5A11 and LINC01608 in OL subtypes of Jäkel et al. [22]. h Violin plots showing expression of OL subtype markers of Jäkel et al. [22] in all four OL subtypes. Suppl. Fig. 9 Transcription factor activity and trajectory analysis of white matter oligodendrocytes in MS. a, b Heatmap showing the altered TF activity in SLC5A11 oligodendrocytes between control and MS cells for cerebrum (a) and spinal cord (b). c Venn diagram showing the overlap of altered TFs identified in (a, b). Notably, almost one fourth of identified TFs overlap between the regions in MS, whereas only five overlaps in control. d Upper panel: UMAP representation of SLC5A11-expressing (SLC5A11+) leukocortical oligodendrocytes. CTRL, control, ACA, acute/chronic-active, CI, chronic-inactive. Lower panel: Trajectory inference of leukocortical SLC5A11+ oligodendrocytes identifies two transcriptomic branches according to inflammatory stage. Note coloring indicates pseudotime score; gray cells do not have a value for one lineage. e Heatmap representation of differentially expressed genes based on pseudotime analysis. Note genes classified as differentially expressed (adjusted p < 0.05) between the endpoints of the two branches (d) are plotted for all SLC5A11+ OLs from both leukocortical and spinal cord MS samples. (PDF 26043 KB)

Suppl. Table 1 Clinical and pathological metadata of control and MS samples (XLSX 15 KB)

Suppl. Table 2 Cluster-specific marker genes for integrated and regional data sets (XLSX 2317 KB)

Suppl. Table 3 Differential gene expression analysis of neuroglial subtypes (XLSX 31 KB)

Suppl. Table 4 Differential expression of region-specific genes in control astrocytes (XLSX 222 KB)

Suppl. Table 5 Enrichment of region-specific GO terms in control astrocytes (XLSX 596 KB)

Suppl. Table 6 Ligand–receptor analysis of Bergmann glia and Purkinje cells (XLSX 64 KB)

Suppl. Table 7 Differential expression of region-specific genes in MS astrocytes (XLSX 66 KB)

Suppl. Table 8 Enrichment of region-specific GO terms in MS astrocytes (XLSX 103 KB)

Suppl. Table 9 Cluster-specific marker genes for integrated astrocytes (XLSX 271 KB)

Suppl. Table 10 Differential gene expression analysis for white matter astrocyte subtypes (XLSX 243 KB)

Suppl. Table 11 Differential expression of region-specific genes in control oligodendrocytes (XLSX 12 KB)

Suppl. Table 12 Differential expression of region-specific genes in control microglia (XLSX 27 KB)

Suppl. Table 13 Differential expression of region-specific genes in control OPCs (XLSX 14 KB)

Suppl. Table 14 Cluster-specific marker genes for integrated oligodendrocytes (XLSX 420 KB)

Suppl. Table 15 Differential gene expression and GO term semantic similarity analysis of SLC5A11-expressing white matter oligodendrocytes from cerebrum and spinal cord (XLSX 49 KB)

Suppl. Table 16 Differential gene expression analysis between trajectory endpoints for subcortical white matter oligodendrocytes (XLSX 40 KB)

## Data Availability

All snRNA-seq data sets (fastq files) were deposited to the Sequence Read Archive (SRA) under accession number PRJNA726991. We have also uploaded the data sets to an interactive web browser for analysis of cell-type-specific expression levels in MS versus control tissues (https://ms-cross-regional.cells.ucsc.edu).
