# Supplementary figures and images for: Cross-regional homeostatic and reactive glial signatures in multiple sclerosis
Source: Acta Neuropathol. 2022 Sep 16;144(5):987–1003. doi: 10.1007/s00401-022-02497-2 (PMC9547805; doi:10.1007/s00401-022-02497-2)

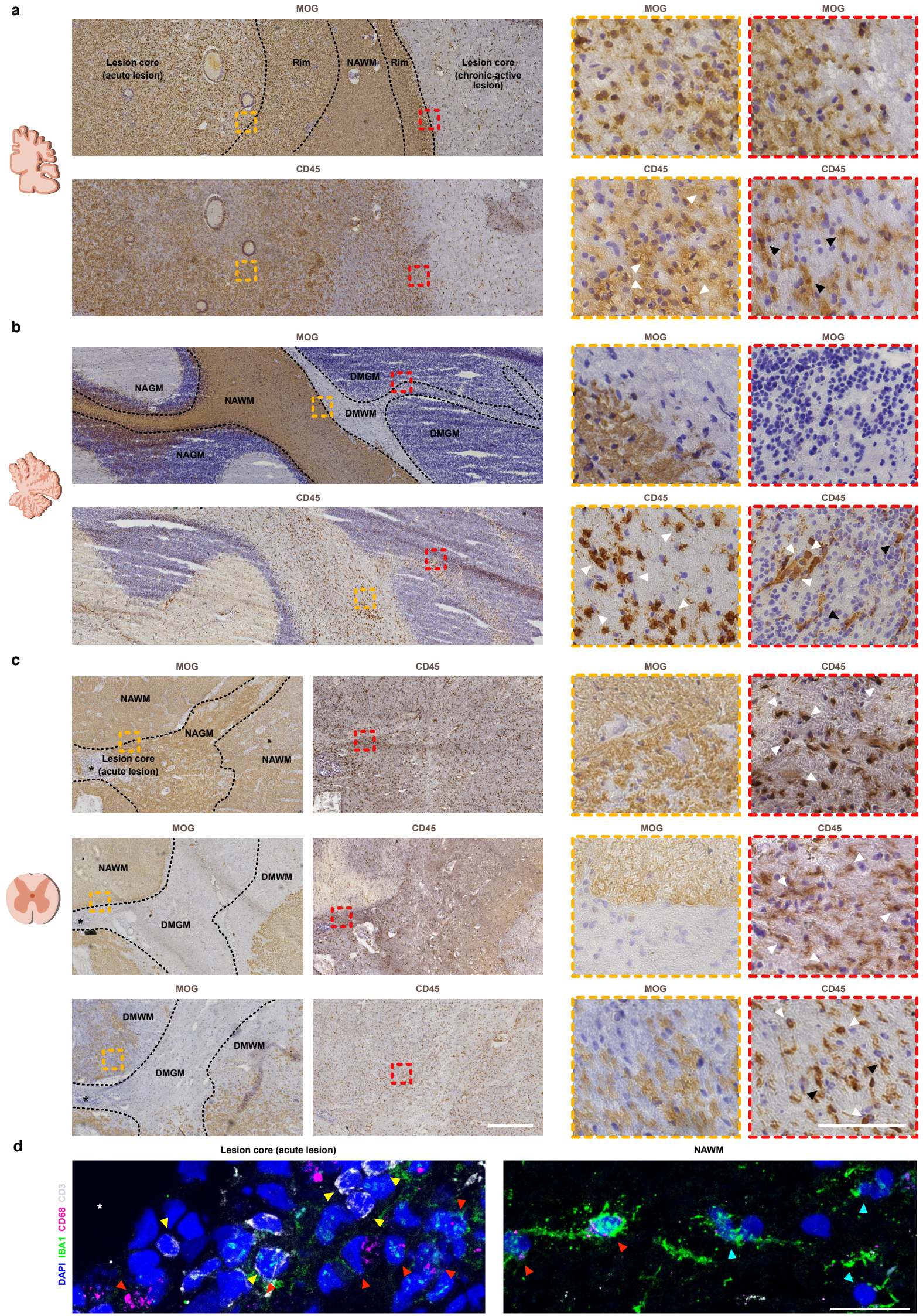

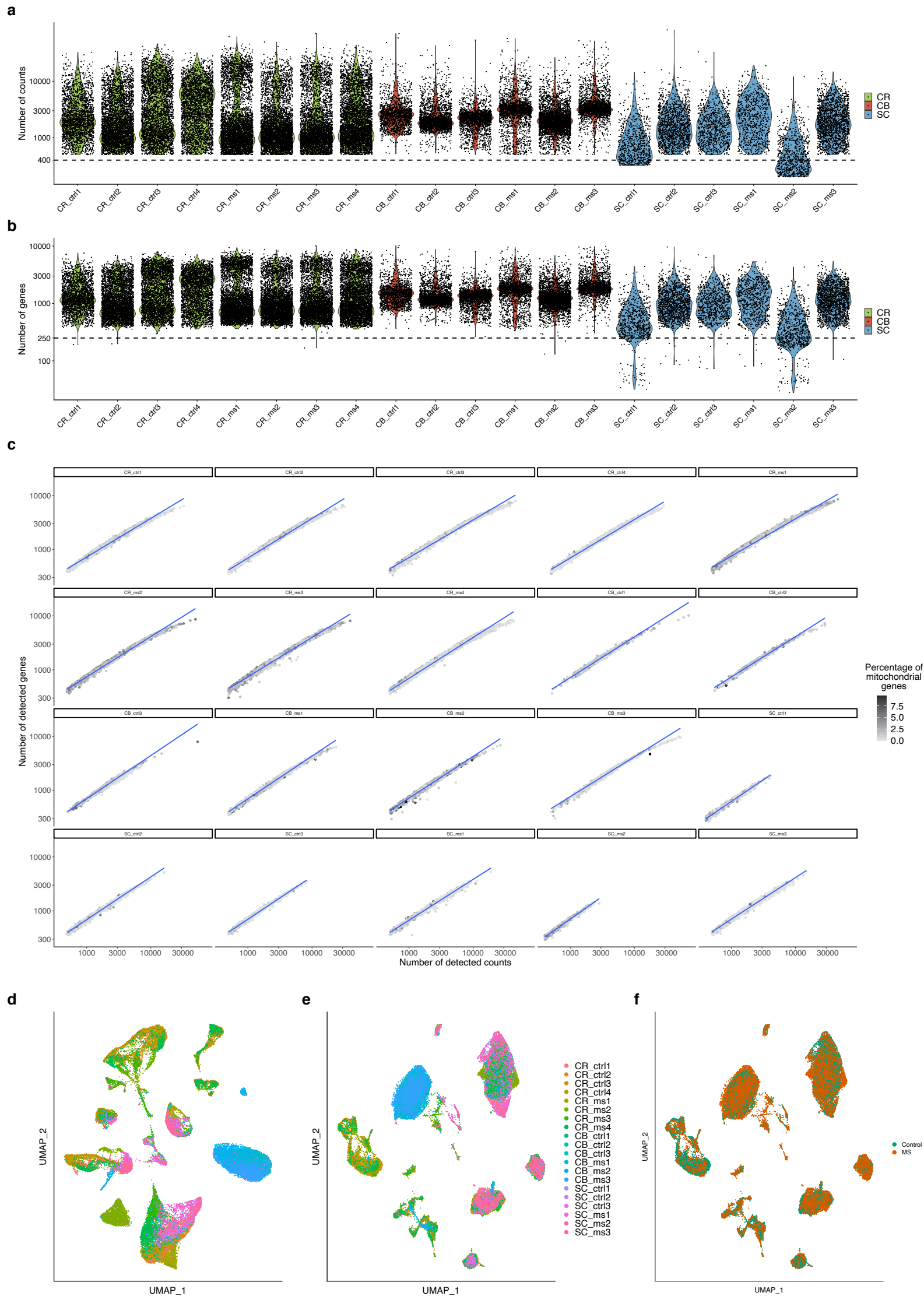

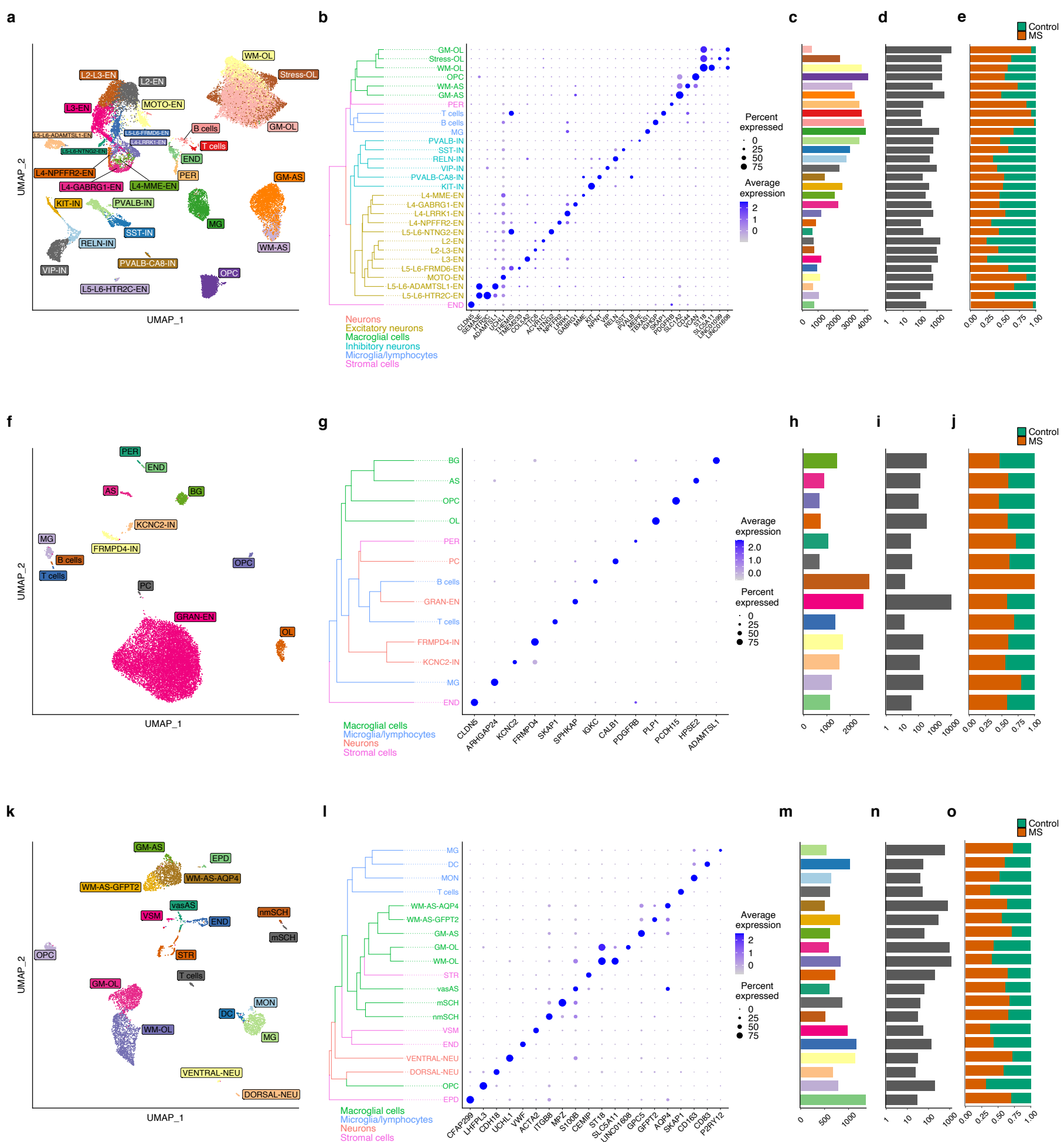

**a**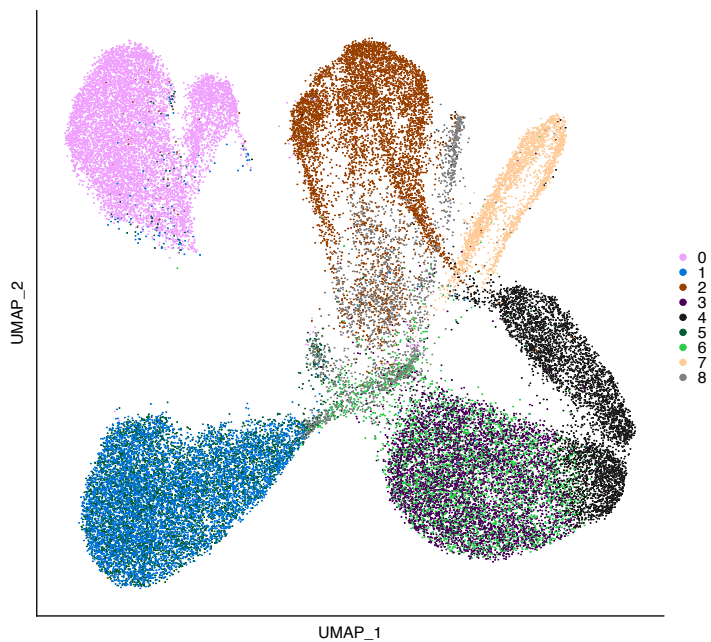**b**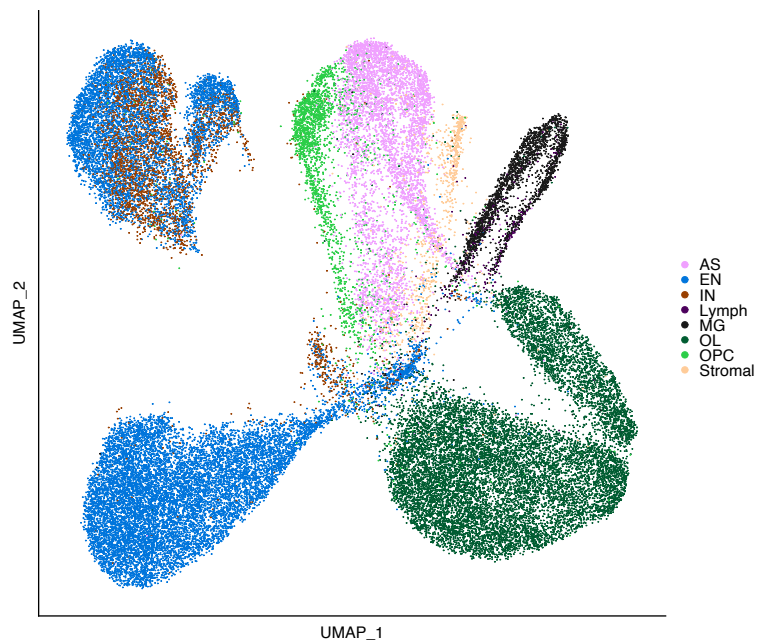**c**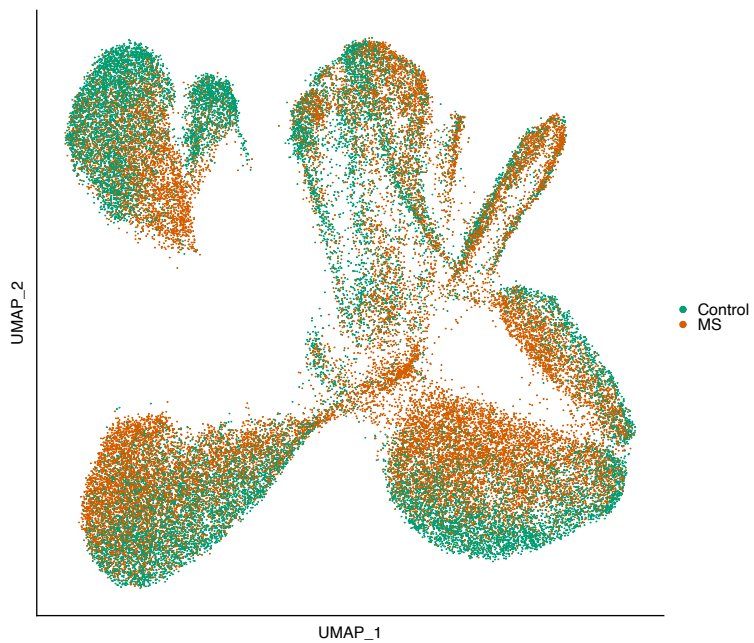**d**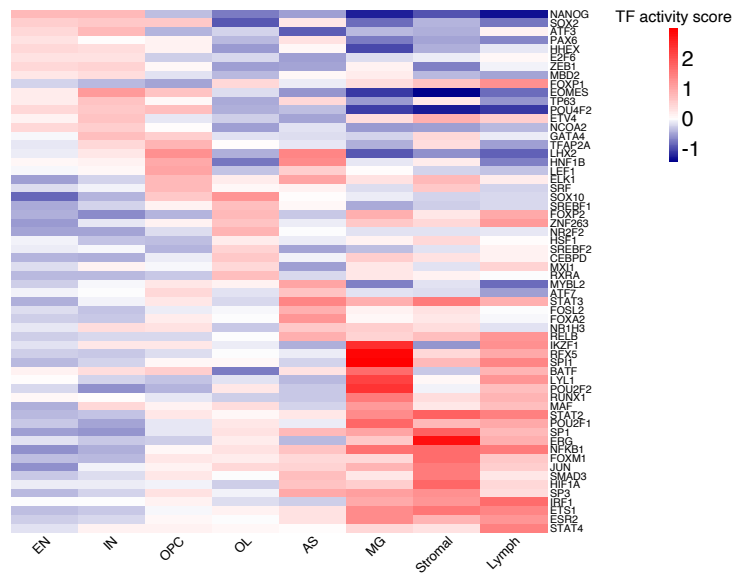

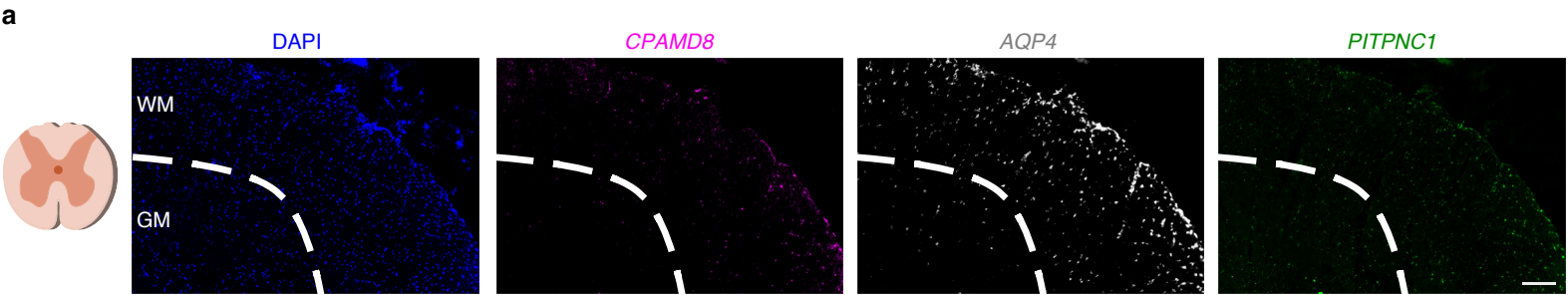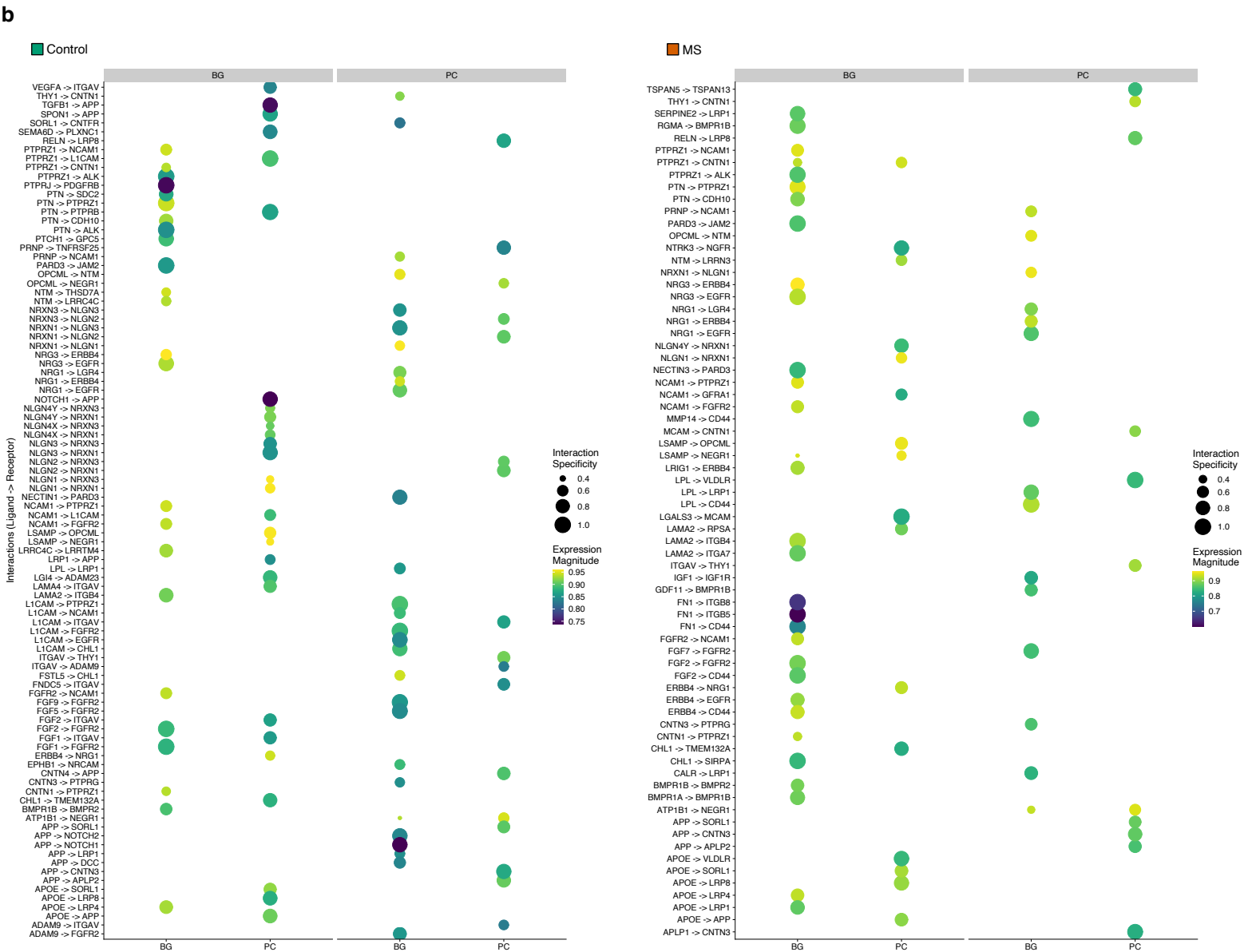

**a**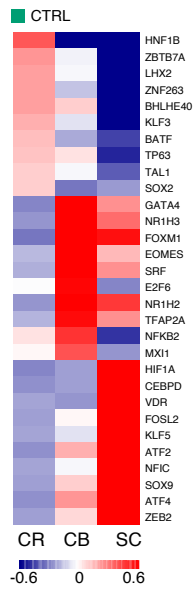**b**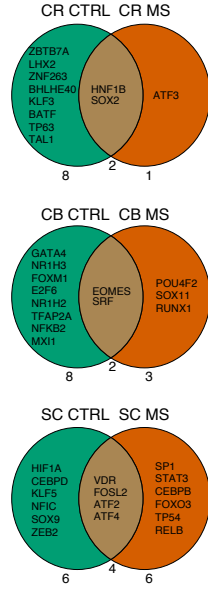**c**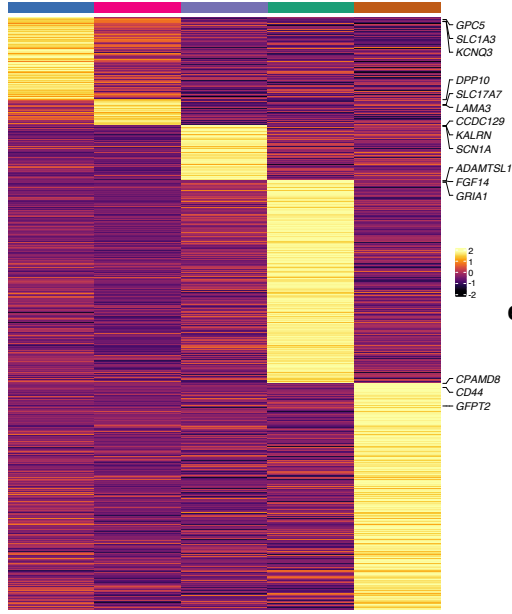**d**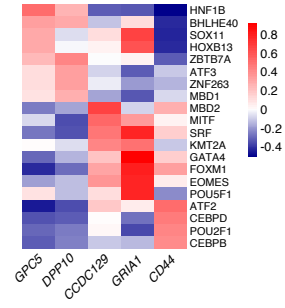**e**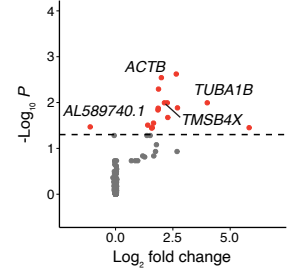

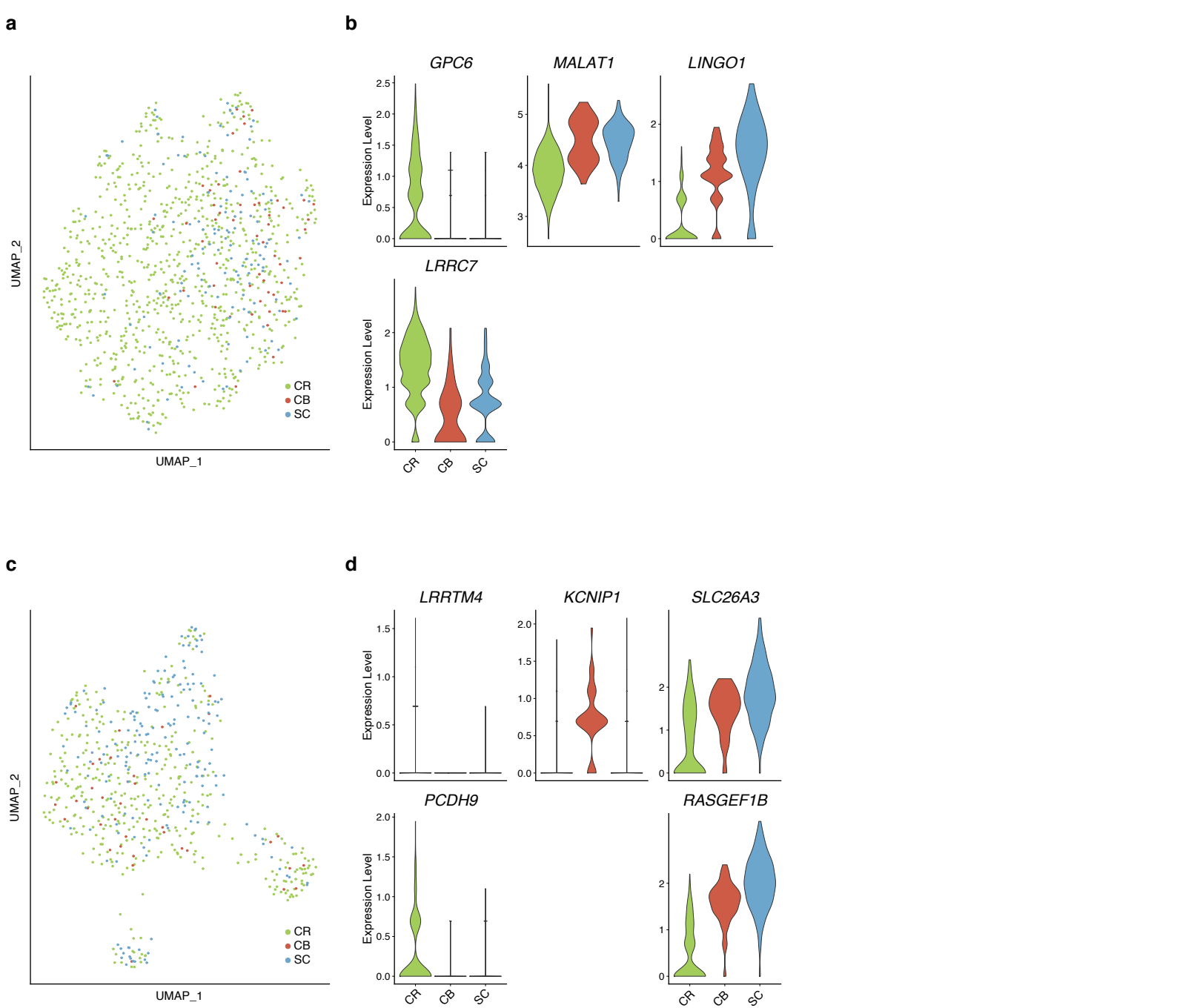

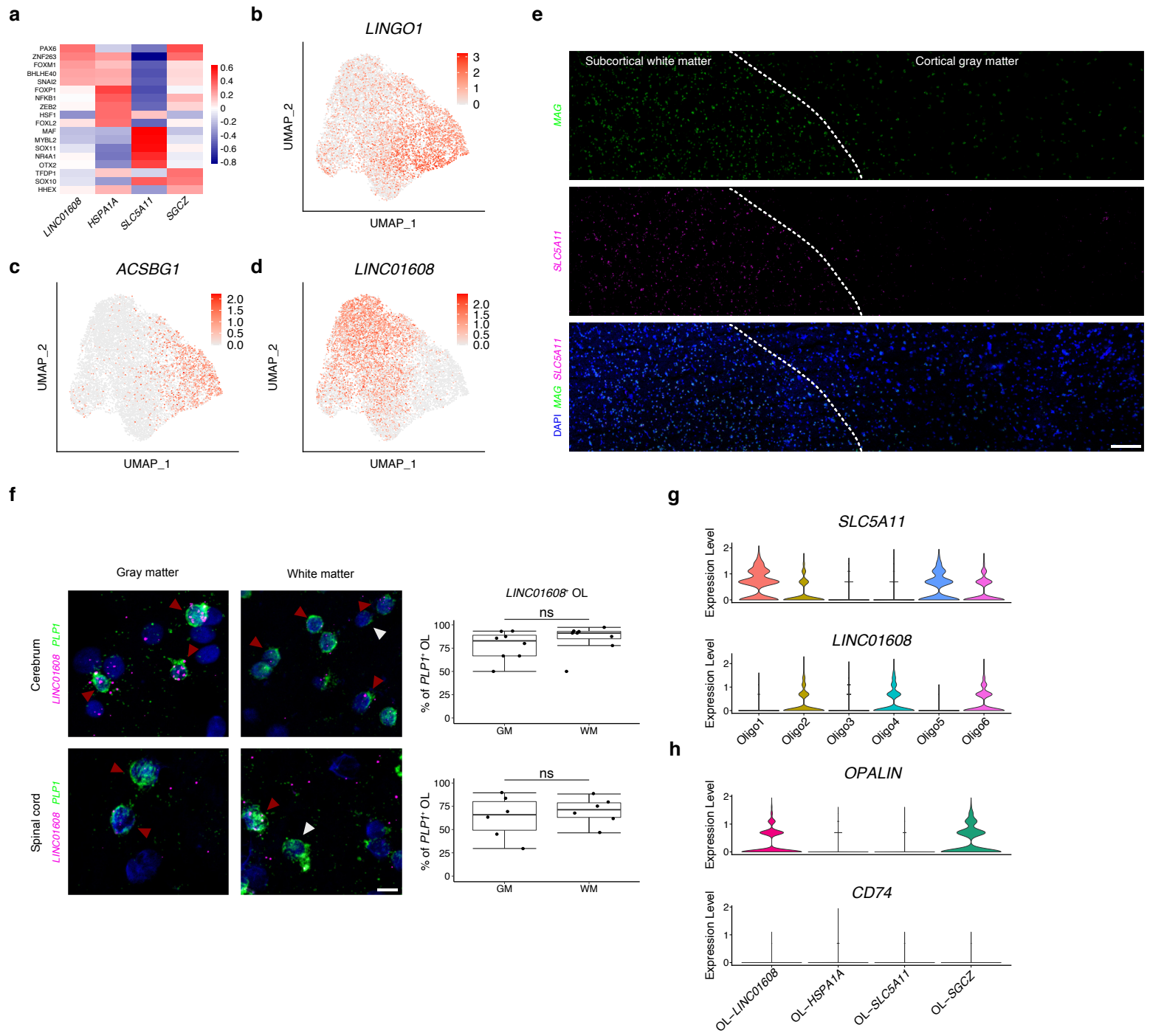

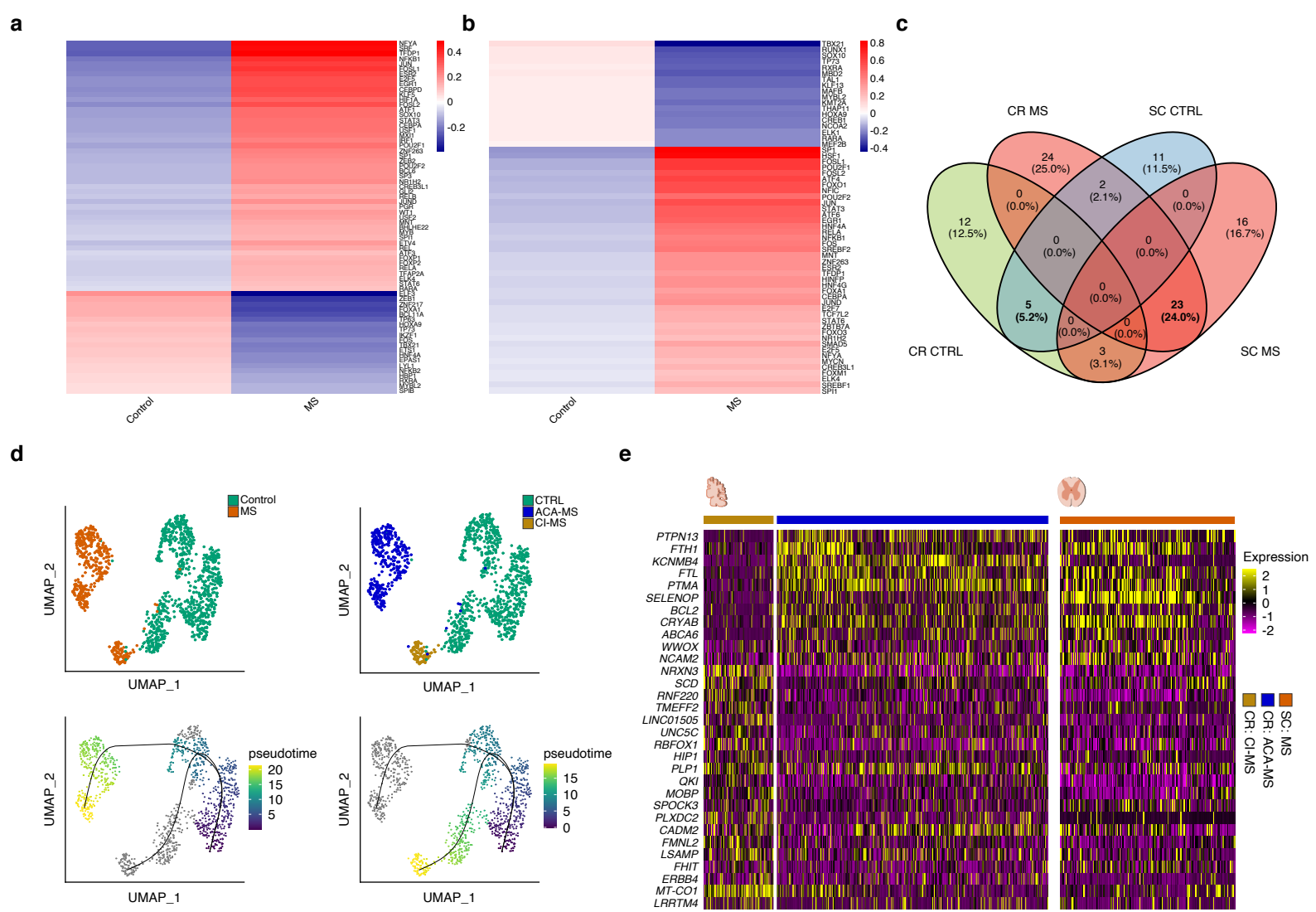

Supplement: Supplementary file 1 — Suppl. Fig. 1 Histological assessment of MS lesions. a MOG and CD45 immunohistochemistry of a representative subcortical MS lesion tissue section. An acute lesion with active demyelination with presence of foam cells (white arrowheads) in the lesion center and rim area is shown next to a chronic-active lesion with demarcated core and hypercellular lesion rim with high levels of microglia (black arrowheads). Overview images for MOG and CD45 were taken on corresponding MS lesion areas of serial sections. b MOG and CD45 immunohistochemistry for a representative cerebellar MS lesion tissue section. A chronic-active cerebellar lesion with demarcated core and hypercellular lesion rim with presence of macrophages (white arrowheads) and microglia (black arrowheads) is shown. Overview images for MOG and CD45 were taken on corresponding MS lesion areas of serial sections. c MOG and CD45 immunohistochemistry for representative spinal cord MS lesion tissue sections. Upper row shows acute spinal cord MS lesion with active demyelination and presence of macrophages (white arrowheads). Lower two rows show chronic-active lesions with demarcated lesion cores in gray and white matter areas with hypercellular lesion rims and presence of few macrophages (white arrowheads) and abundant microglia (black arrowheads). Overview images for MOG and CD45 were taken on corresponding MS lesion areas of serial sections. Scale bars indicate 500 µm on overview images and 100 µm on zoom ins. d Fluorescence immunohistochemistry for IBA1, CD68 and CD3. On the left side, NAWM with macrophages (IBA1+/CD68+, red arrowheads) and resident ramified microglia (CD68+, cyan arrowheads) is shown. On the right side, image displays a lesion core of an acute lesion. T cells (CD3+, yellow arrowheads) are shown next to tissue-infiltrating macrophages (IBA1+/CD68+, red arrowheads) coming from a nearby vessel (asterisk). Scale bar indicates 20 µm. Suppl. Fig. 2 Quality control and batch correction of snRNA-seq data. a [file 401_2022_2497_MOESM1_ESM.pdf]
